# Dysbindin-1 regulates mitochondrial fission and gamma oscillations

**DOI:** 10.1038/s41380-021-01038-9

**Published:** 2021-02-15

**Authors:** Jun Zhao, Huiwen Zhu, Kaizheng Duan, Ronald S. Petralia, Ya-Xian Wang, Qinhua Gu, Debabrata Panja, Zheng Li

**Affiliations:** 1 Section on Synapse Development Plasticity, National Institute of Mental Health, National Institutes of Health, Bethesda, Maryland United States; 2 Advanced Imaging Core, National Institute on Deafness and Other Communication Disorders, National Institutes of Health, Bethesda, Maryland United States

**Keywords:** Neuroscience, Cell biology

## Abstract

Mitochondria are cellular ATP generators. They are dynamic structures undergoing fission and fusion. While much is known about the mitochondrial fission machinery, the mechanism of initiating fission and the significance of fission to neurophysiology are largely unclear. Gamma oscillations are synchronized neural activities that impose a great energy challenge to synapses. The cellular mechanism of fueling gamma oscillations has yet to be defined. Here, we show that dysbindin-1, a protein decreased in the brain of individuals with schizophrenia, is required for neural activity-induced fission by promoting Drp1 oligomerization. This process is engaged by gamma-frequency activities and in turn, supports gamma oscillations. Gamma oscillations and novel object recognition are impaired in dysbindin-1 null mice. These defects can be ameliorated by increasing mitochondrial fission. These findings identify a molecular mechanism for activity-induced mitochondrial fission, a role of mitochondrial fission in gamma oscillations, and mitochondrial fission as a potential target for improving cognitive functions.

## Introduction

Mitochondria are vital organelles in eukaryotic cells. Neuronal mitochondria are motile and undergo fission and fusion. Mitochondrial fission is mediated by the dynamin-related GTPase Drp1. During mitochondrial fission, cytosolic Drp1 is recruited to mitochondria, binds to adaptors on outer mitochondrial membranes, and assembles into oligomers to constrict and sever mitochondrial membranes [1]. The ratio of mitochondrial fission to fusion is increased by neural depolarization [2].

Mitochondria generate ~90% of cellular ATP in neurons [3, 4]. They are essential for synaptic transmission, an energy-demanding process accounting for 41% of ATP consumed in the rat cortex and even more in the primate cortex [3, 5, 6]. A neuron’s energy expenditure is proportional to the frequency of synaptic transmission [3]. Neural activities at higher frequencies are more mitochondria-dependent [7].

Gamma oscillations are high-frequency (~20–100 Hz), synchronized activities of neural populations that require strong mitochondrial functions [7, 8]. They are present in many brain areas and underlie the precise timing of neuronal discharges and coherent binding of neural ensembles for information processing during cognitive functions [9, 10]. The mechanism for generating gamma oscillations is best characterized in the hippocampal CA3 region where both excitatory and inhibitory synaptic transmissions are required for gamma oscillations, and the oscillating local field potentials (LFPs) mainly originate from perisomatic inhibitory currents in pyramidal neurons [11–15]. The power of gamma oscillations is dependent on the ability of excitatory neurons to rapidly repolarize postsynaptic membranes after each synaptic input. Pumping out ions flowing into excitatory postsynaptic neurons to repolarize membrane potentials accounts for ~50% of ATP used for synaptic transmission [3]. Despite the recognized importance of mitochondria in gamma oscillations, little is known about how mitochondria accommodate the rapid, local increase in energy demands imposed by gamma oscillations at the postsynaptic site of excitatory neurons.

Dysbindin-1 is a coiled-coil domain-containing protein decreased in the brains of people with schizophrenia [16]. It regulates the subcellular distribution of dopamine D2, D3 receptors, the NMDA receptor subunit GluN2A, necdin, and BDNF, as well as glutamate release, neural excitability, and synaptic plasticity [16–21]. Dysbindin-1 is present on mitochondrial outer membranes [22]. It is unknown, however, whether dysbindin-1 affects mitochondria.

In this study, we show that dysbindin-1 regulates mitochondrial fission by promoting Drp1 oligomerization on mitochondria. Neuronal activation in the gamma band induces the translocation of dysbindin-1 to mitochondria where it interacts with Drp1 and the Drp1 receptor Mid49 and Mid51 to increase Drp1 oligomerization and mitochondrial fission. This process is required for gamma oscillations. Dysbindin-1 null mice (sdy) have reduced mitochondrial fission, which leads to deficits in gamma oscillations and novel object recognition (NOR). Notably, these deficits can be alleviated by increasing mitochondrial fission with a light-inducible mitochondrial fission system. These findings reveal an unsuspected role of dysbindin-1 in mitochondrial dynamics and gamma oscillations.

## Materials and methods

Methods, key reagents, and software are described in more detail in Supplementary information.

### Animals

All animal procedures followed the NIH Guidelines Using Animals in Intramural Research and were approved by the National Institute of Mental Health Animal Care and Use Committee. Sdy mice (backcrossed to the C57BL/6J background for >10 generations) and wild-type (WT) mice (C57BL/6) were purchased from the Jackson Laboratory. Male animals were used for behavioral and in vivo electrophysiology.

### Neural culture, glia culture, and transfection

Primary hippocampal neurons were prepared from E18-19 rat embryos and transfected as previously described [23]. For glial cultures, dissociated hippocampal cells from mouse embryos (E18-19) were cultured in DMEM medium.

### Hippocampal slices

Organotypic hippocampal slices were cultured from WT or sdy mice (6–8 days of age) as described in [24]. For acute hippocampal slices, mice were anesthetized by isoflurane overdose. The brain was removed, submerged in ice-cold cutting buffer and cut into 400-μm thick horizontal brain slices with a Leica VT1000S vibratome.

### Electrophysiology

Field potentials of hippocampal slices were recorded in an interface recording chamber maintained at 28–32 °C. Recording electrodes were placed at the stratum pyramidale of the CA3 region ~200 μm below the surface. Whole-cell recordings were performed in a submerged chamber perfused with ACSF at a rate of 2 ml/min. The electrical signals were amplified with Axon Multiclamp 700B and digitized at 10 kHz with Axon Digidata 1440A. In vivo recording began at 1 week after surgery by using an Intan 32-channel RHD2000 evaluation system.

### Live imaging

Cultured hippocampal neurons or hippocampal slices were placed in a chamber mounted on the sample stage of an Olympus FV1000 confocal microscope, perfused with ACSF at a rate of 2 ml/min at 30 °C. Images were acquired with a 60X objective (NA = 1.0) every 15 s.

### NOR test

The NOR test had two sessions, a sample session and a test session. The two sessions were separated by 6 h. The second NOR test was conducted 1 week after the first NOR test.

### Surgery

Mice were anesthetized with isoflurane. 1 μl lentivirus was injected into the CA3 region. For electrophysiological recording in hippocampal slices, the following coordinates were used: AP, −2 mm; ML, ±1.9 mm; DV, −1.8–1.5 mm. Optic fibers and microdrive-controlled optoelectrodes were implanted 100 μm above the viral injection site.

## Results

### Dysbindin-1 restricts mitochondrial length

To test whether dysbindin-1 regulates organelles, we examined mitochondria in sdy mice, which carry a deletion mutation in the dysbindin-1 gene. Since dysbindin-1 is expressed in glutamatergic but not in GABAergic neurons of the hippocampus [25, 26], we transduced cultured hippocampal slices (7 days in vitro, DIV7) prepared from WT and sdy mice with lentivirus expressing mitoDsRed (mitochondria-targeted DsRed) driven by the CaMKIIα promoter and Venus (for visualization of neuronal morphology). Confocal images of hippocampal pyramidal neurons which are glutamatergic were acquired 4–6 days later. We considered mitochondria within thick processes populated with dendritic spines as dendritic and those in thin processes without dendritic spines to be axonal. We confirmed that neuronal processes with dendritic spines were dendrites as they contained the dendritic marker MAP2, and that smooth, thin neuronal processes without dendritic spines were axons as they were positive for the axonal marker Tau and negative for MAP2 (Supplementary Fig. 1).

**Fig. 1:**
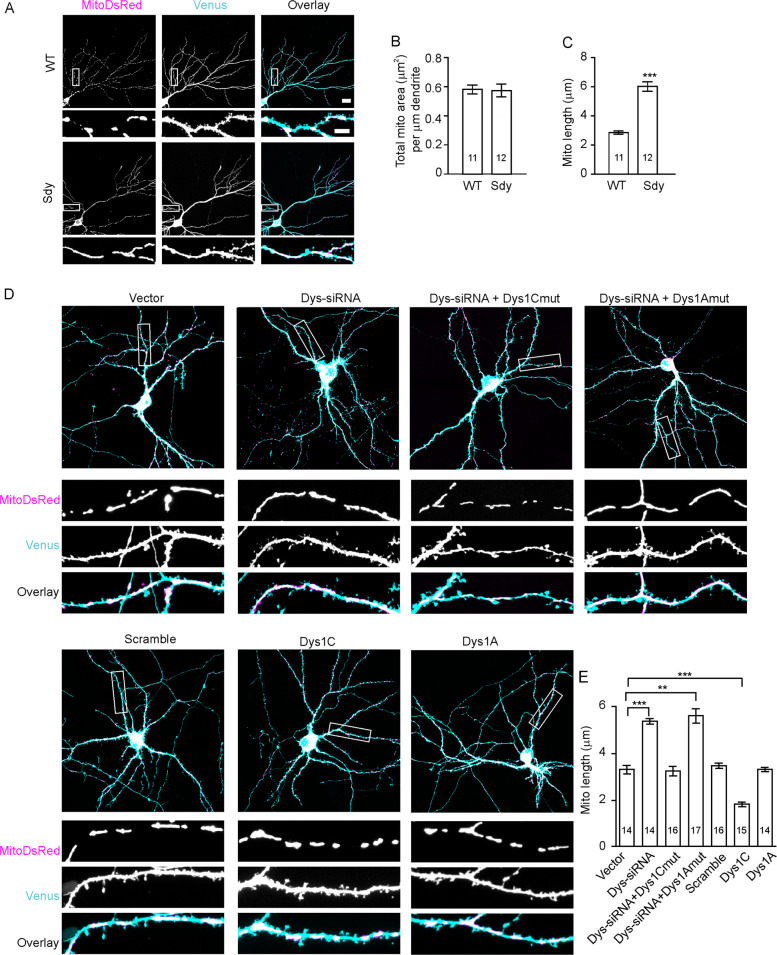
Dysbindin-1 controls mitochondrial length. Hippocampal slices cultured from sdy mice and their WT littermates (**A**–**C**) or cultured WT hippocampal neurons (**D**, **E**), were used for confocal imaging of mitochondria. **A** Representative images of mitochondria in the CA3 region; scale bar, 10 μm. **B**, **C** Quantification for **A**; Mann–Whitney *U* test (**B**) and Student’s *t*-test (**C**) were used; n in the bars indicates the number of brain slices from four WT and five sdy mice. **D** Representative images of transfected neurons; scale bar, 20 μm for upper and 5 μm for lower, enlarged images. **E** Quantification for **D**; one-way ANOVA on ranks was used to compare across groups (*H* = 82.78, DF = 6, *p* < 0.001); Student’s *t*-test was used for vector vs. Dys-siRNA, vector vs. Dys-siRNA + Dys1Cmut, vector vs. Dys-siRNA + Dys1Amut; Mann–Whitney *U* test was for vector vs. scrambled, vector vs. Dys1C, vector vs. Dys1A; n in the bars indicates the number of neurons. Data are presented as mean ± SEM. **p < 0.01, ***p < 0.001.

In hippocampal slices, dendritic mitochondria had a sufficiently high density and elongated shape that aligned with the dendritic trajectory to allow for assigning mitochondria to dendrites based on mitoDsRed imaging. However, the low density and less elongated shape of axonal mitochondria and the frequent crossing of axons and dendrites made the assignment of axonal mitochondria difficult. We, therefore, focused on dendritic mitochondria in hippocampal slices. While total mitochondrial area was comparable in sdy and WT slices (two-tailed Student’s *t*-test, *p* = 0.898), mitochondria were longer in sdy slices (WT: 2.85 ± 0.10 μm; sdy: 6.02 ± 0.32 μm; Mann–Whitney *U* test, *p* = 0.001; Fig. 1A–C).

To avoid the non-specific effect of chronic dysbindin-1 deletion and confirm our findings using a different preparation, we transfected cultured hippocampal neurons with a plasmid expressing siRNAs against dysbindin-1 along with the mitoDsRed and Venus plasmids at DIV14. The specificity and efficacy of the dysbindin-1 siRNA have been validated in our previous studies [27]. We analyzed mitochondria at DIV17 in spiny neurons, which are excitatory in the hippocampus. Dendritic mitochondria were longer in dysbindin-1 siRNA but not in scrambled siRNA-transfected neurons (vector control: 3.32 ± 0.18 μm; dysbindin siRNA: 5.39 ± 0.11 μm, *p* < 0.001 vs. control, Mann–Whitney *U* test; scrambled siRNA, 3.48 ± 0.10 μm, *p* = 0.42 vs. control, two-tailed Student’s *t*-test; Fig. 1D, E). These findings are consistent with those in sdy cells.

Dysbindin-1 has two splicing isoforms in rodents: dysbindin-1A (full-length) and dysbindin-1C (N-terminal truncated) [28], both of which are deleted in sdy mice and targeted by the dysbindin-1 siRNA [27]. To test which isoform regulates mitochondrial length, we co-transfected cultured hippocampal neurons (DIV14) with the dysbindin-1 siRNA construct and plasmids expressing dysbindin-1A or -1C resistant to dysbindin-1 siRNAs due to synonymous mutations at the dysbindin-1 siRNA binding site (dys1Cmut and dys1Amut) [27]. In the dendrite of dysbindin-1 knockdown cells, mitochondrial length was restored by dys1Cmut, and dysbindin-1C overexpression shortened mitochondria (dysbindin-1 siRNA plus dys1Cmut: 3.25 ± 0.21 μm, *p* = 0.784 vs. control; dysbindin-1C, 1.82 ± 0.09 μm, *p* = 9.3 × 10^−8^ vs. control; two-tailed Student’s *t*-test; Fig. 1D, E). By contrast, dysbindin-1A overexpression had no effect on mitochondrial length or changed the effect of dysbindin-1 siRNAs (dysbindin-1 siRNA plus dys1Amut: 5.63 ± 0.27 μm, *p* = 1.49 × 10^−7^ vs. control; dysbindin-1A: 3.32 ± 0.09 μm, *p* = 0.977 vs. control; two-tailed Student’s *t*-test; Fig. 1D, E). These results indicate that loss of dysbindin-1C, but not dysbindin-1A, causes mitochondrial elongation.

To assess axonal mitochondria, we transfected primary hippocampal neurons from WT and sdy mice with plasmids expressing mitoDsRed and Venus at DIV14 and took confocal images 3 days later. The medium cell density in primary cultures allowed for tracing of axons which were readily distinguished from dendrites by their small diameters and lack of dendritic spines. The average length of axonal mitochondria was unchanged in sdy or dysbindin-1 siRNA-transfected neurons (Supplementary Fig. 2A–D). Mitochondrial length was also comparable in WT and sdy glia (Supplementary Fig. 2E, F).

Moreover, we assessed mitochondrial motility with time-lapse imaging in hippocampal slices and primary hippocampal neurons. Mitochondria with a net displacement of greater than or equal to their length during the imaging period were identified as motile mitochondria. The proportion and speed of motile mitochondria were comparable in WT and sdy slices, in WT and sdy primary hippocampal neurons, and were unchanged by dysbindin-1 siRNAs (Supplementary Fig. 3). Hence, dysbindin-1 deficiency does not affect mitochondrial motility.

Taken together, these findings indicate that dysbindin-1C restricts mitochondrial length.

### Dysbindin-1 regulates mitochondrial fission

Mitochondrial length is dependent on the balance of mitochondrial fission and fusion. To determine the mechanism responsible for mitochondrial elongation in sdy mice, we assessed mitochondrial fission and fusion in cultured WT and sdy slices. Slices were transduced with lentivirus expressing mitoDsRed at DIV7. At DIV10–12, slices were transferred to a chamber mounted on a confocal microscope, perfused with artificial cerebrospinal fluid (ACSF, 37 °C, bubbled with 95% O_2_/5% CO_2_) for 1 h and then imaged every 15 s for 60 min. Fission was identified from mitochondrial division, and fusion was defined as the coalescing of two mitochondria into one that stayed as one structure for ≥1 min.

**Fig. 2:**
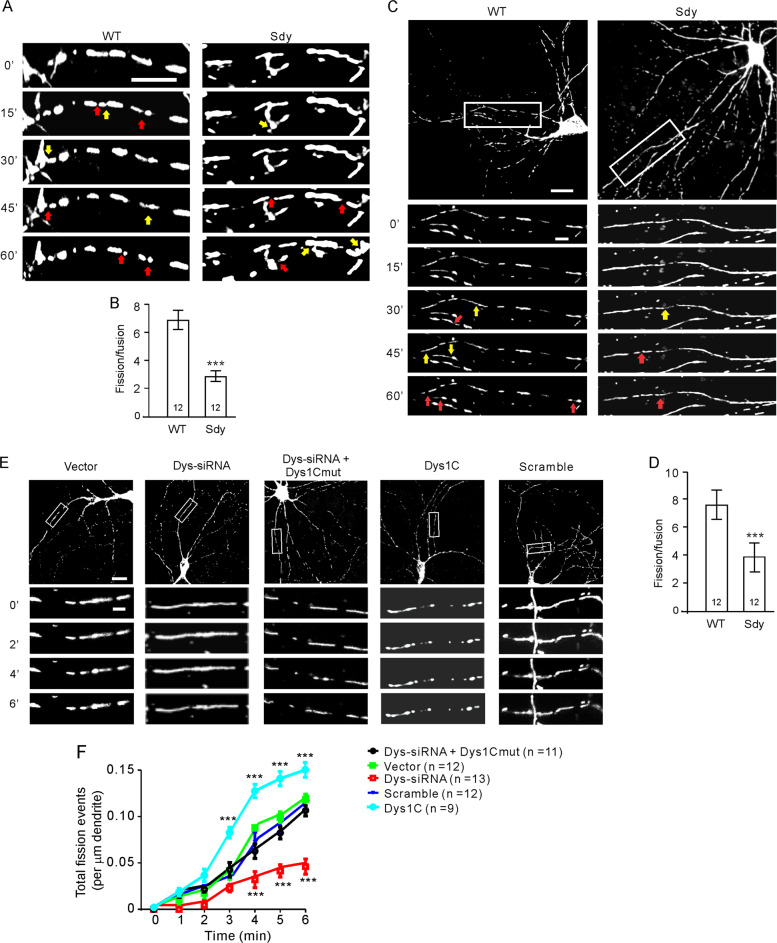
Mitochondrial fission is impaired in dysbindin-1 deficient neurons. Hippocampal slices cultured from sdy mice and their WT littermates (**A**, **B**) or cultured hippocampal neurons (**C**–**F**) were used for time-lapse imaging of mitochondria. **A** Representative time-lapse images of mitochondria in the CA3 region; scale bar, 5 μm. **B** Quantification for **A**; the fission and fusion rates were defined as the number of fission or fusion events identified within 10 μm dendrite per min; Mann–Whitney *U* test was used for statistical analysis; n in the bars indicates the number of brain slices from four animals. **C** Representative time-lapse images of mitochondria in primary WT and sdy hippocampal neurons; scale bar, 20 μm for upper and 5 μm for lower, enlarged images. **D** Quantification for **C**; Mann–Whitney *U* test was used for statistical analysis; n in the bars indicates the number of neurons. **E** Representative images of mitochondria in transfected neurons at designated time points after photostimulation; scale bar, 20 μm for upper and 5 μm for lower, enlarged images. **F** Quantification of the number of total fission events identified within 10 μm dendritic segments after photostimulation for **E**; two-way RM ANOVA was used to test for the influence of treatment on fission, *p* < 0.001; Holm-Sidak test was used for comparisons of Dys-siRNA vs. vector, vector vs. scrambled, Dys-siRNA + Dys1cmut vs. vector; Mann–Whitney *U* test was used to compare Dys-siRNA vs. vector and Dys1C vs. vector at different time points; *n* = 13 neurons for Dys-siRNA, 12 neurons for vector, 12 neurons for scrambled siRNAs, and 11 neurons Dys-siRNA + Dys1cmut. Mitochondrial fission sites were indicated by red arrows and mitochondrial fusion sites with yellow arrows in **A**, **C**. Data are presented as mean ± SEM; ***p < 0.001. (color figure online).

The ratio of mitochondrial fission to fusion rate was ~1 in both WT and sdy slices (Supplementary Fig. 4A, B). However, during the first hour after slices had been transferred to the imaging chamber when they were adapting to the change from culture media to ACSF, the fission to fusion ratio increased, and this increase was more in WT than in sdy slices (Fig. 2A, B). Likewise, in primary hippocampal neurons, while the fission to fusion ratio was ~1 after neurons had been transferred to the imaging chamber for >1 h in both WT and sdy neurons (Supplementary Fig. 4C, D), it was lower in sdy neurons than in WT neurons within 1 h after the transfer (Fig. 2C, D).

The increase in mitochondrial fission to fusion ratio shortly after transfer can be due to transfer-induced neural activation which has been shown to induce mitochondrial fission [2, 29]. The lower fission to fusion ratio in sdy neurons post transfer suggests that dysbindin-1 may be involved in activity-induced mitochondrial fission. To test this possibility, we co-transfected cultured hippocampal neurons (DIV14) with the dysbindin-1 siRNA, mitoDsRed, and channelrhodopsin-2 (ChR2, for light-induced neural activation) constructs, and depolarized neurons with light pulses. We chose this method because light illumination in single-layered cultures allows even stimulation of the entire neuron with precise temporal control.

At 3 days after transfection, neurons were illuminated at 473 nm (40 Hz, 1 ms pulse duration, 2 mW/mm^2^) for 6 min while mitochondria were imaged every 15 s. 40-Hz light stimulation was used because our electrophysiological recordings showed that hippocampal pyramidal neurons can fire action potentials with high fidelity in response to a train of electrical pulses up to 40 Hz (Supplementary Fig. 4E). We confirmed that 40-Hz light pulses depolarize cultured hippocampal neurons as indicated by light-evoked excitatory postsynaptic currents (Supplementary Fig. 4F).

Light stimulation increased mitochondrial fission (Fig. 2E, F). The total fission events induced by illumination, however, were reduced by dysbindin-1 siRNAs, but not by scrambled siRNAs (Fig. 2E, F). The effect of dysbindin-1 siRNAs on mitochondrial fission was obliterated by co-transfection of dys1Cmut, while dysbindin-1C overexpression increased light-induced mitochondrial fission (Fig. 2E, F). These findings indicate that dysbindin-1C contributes to neural activity-induced mitochondrial fission.

To test whether dysbindin-1 is also involved in non-neural activity-induced mitochondrial fission, we applied carbonyl cyanide *m*-chlorophenyl hydrazone (CCCP, a protonophore inducing mitochondrial fission by disrupting MMPs) to neurons. CCCP-induced mitochondrial fission was comparable in WT, sdy, and dysbindin-1 siRNA-transfected neurons (Supplementary Fig. 4G, H). These results indicate that dysbindin-1 is not required for fission induced by mitochondrial depolarization, therefore the core mitochondrial fission machinery is functional in dysbindin-1 deficient neurons.

Taken together, these findings indicate that dysbindin-1C regulates neural activity-induced mitochondrial fission.

### Dysbindin-1 promotes Drp1 oligomerization

Mitochondrial fission requires Drp1. Drp1 is a large GTPase predominantly localized in the cytosol and recruited to mitochondria during mitochondrial fission where it forms an oligomeric ring around the mitochondrial tubule to constrict and sever mitochondrial outer membranes [1]. Having found that dysbindin-1 regulates mitochondrial fission, we proceeded to test whether dysbindin-1 is involved in the association of Drp1 with mitochondria by staining Drp1 in primary hippocampal neurons. Primary neurons were used because their single layer and medium-density significantly reduce neuronal process overlap, which is high in brain slices.

**Fig. 3:**
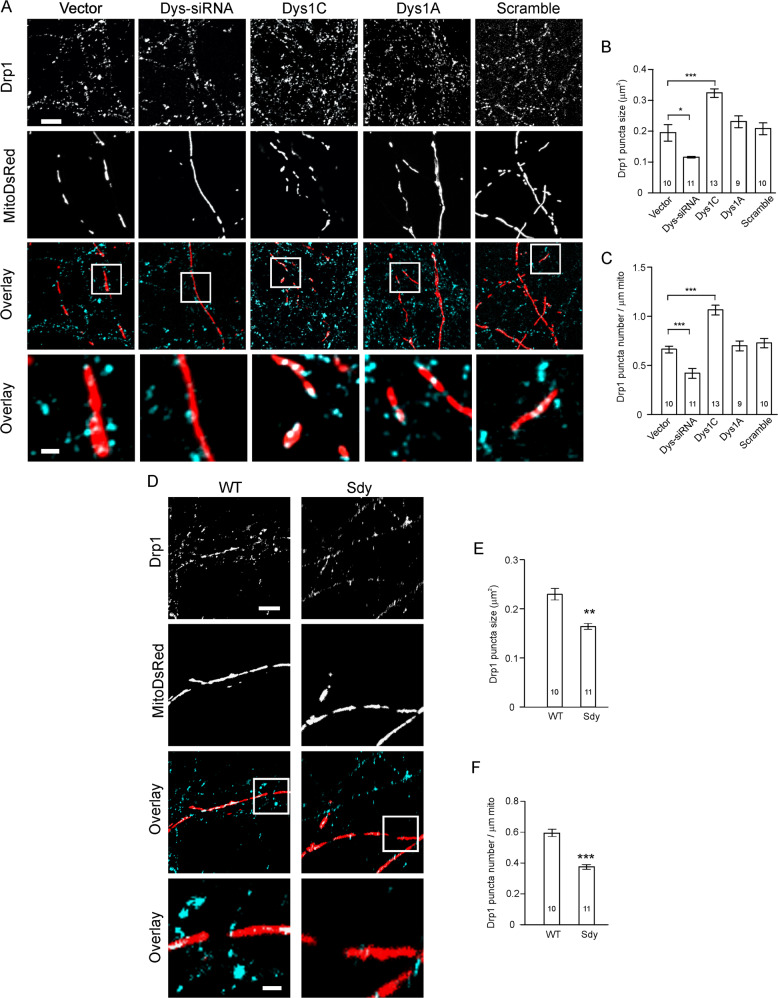
Dysbindin-1 promotes Drp1 accumulation on mitochondria. Cultured hippocampal neurons were transfected with designated plasmids and stained for Drp1. **A**, **D** Representative images; scale bar, 5 μm in **A** and **D** for low-magnification images, 1 μm in **A** and **D** for high-magnification images. **B**, **C** Quantification for Drp1 puncta on mitochondria for **A**; **B** one-way ANOVA on ranks was used to compare across groups (H = 34.444, DF = 4, *p* < 0.001), Mann–Whitney *U* test was used to compare vector vs. Dys-siRNA, Student’s *t*-test was used to compare vector vs. Dys1C, vector vs. Dys1A and vector vs. scrambled; **C** one-way ANOVA was used to compare across groups [*F*_(4, 48)_ = 30.022, *p* = 0.001], and Student’s *t*-test was used for comparison between two conditions. **E**, **F** Quantification for Drp1 puncta on mitochondria in **D**; Mann–Whitney *U* test was used for statistical analysis. 2–4 dendritic segments of 5–10 μm long in each cell were measured; n in the bar indicates the number of cells. Data are presented as mean ± SEM; *p < 0.05, **p < 0.01, ***p < 0.001.

Cultured WT hippocampal neurons (DIV14) were transfected with the mitoDsRed plasmid along with the dysbindin-1 siRNA, scrambled siRNA, dysbindin-1C, or -1A plasmid, and stained with an antibody against Drp1 at DIV17. While the number and size of Drp1 puncta on mitochondria were left intact in dysbindin-1A and scrambled siRNA-transfected cells, they decreased in dysbindin-1 siRNA-transfected cells and increased in dysbindin-1C transfected cells (Fig. 3A–C). Likewise, there were fewer and smaller Drp1 puncta on mitochondria in primary sdy neurons than in WT neurons (Fig. 3D–F).

**Fig. 4:**
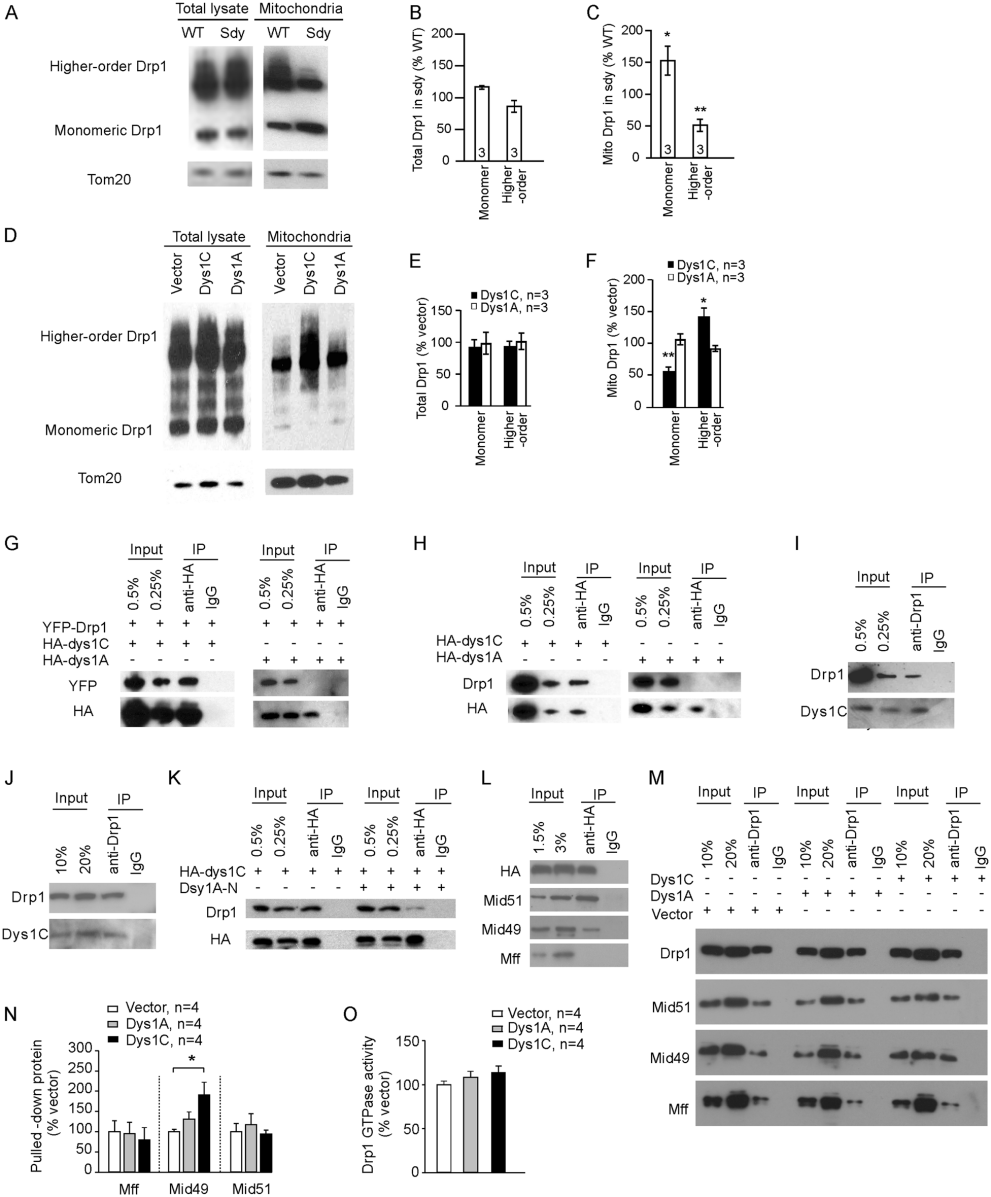
Dysbindin-1 binds to and promotes Drp1 oligomerization. Total lysates and the mitochondrial fraction were prepared from the hippocampus of sdy mice and their WT littermates (**A**–**C**) or cultured hippocampal neurons transduced with designated lentivirus (**D**–**F**), crosslinked and separated by gel electrophoresis. **A**, **D** Represent blots. **B**, **C** Quantification for **A**; Student’s *t*-test was used to compare Drp1 monomer and higher-order structures between sdy and WT mitochondrial fractions; n in the bars indicates the number of mice. **E**, **F** Quantification for **D**; one-way ANOVA was used to compare across groups in **E** [*F*_(4, 10)_ = 0.121, *p* = 0.972] and **F** [*F*_(4, 10)_ = 16.371, *p* < 0.001]; Student’s *t*-test was used to compare Dys1C vs. vector and Dys1A vs. vector for Drp1 monomer and higher-order structures; n indicates the number of neuronal cultures. **G**, **K**, **L** Immunoprecipitation from HEK-293 cells transfected with designated plasmids using the anti-HA antibody. **H** Immunoprecipitation from cultured neurons transduced with designated virus using the anti-HA antibody. **I** Immunoprecipitation from the hippocampus of WT mice using an anti-Drp1 antibody. **J** Immunoprecipitation from cultured WT neurons using an anti-Drp1 antibody. **M** Immunoprecipitation from HEK-293 cells transfected with designated plasmids using an anti-Drp1 antibody. **N** Quantification for **M**; one-way ANOVA was used to compare across groups for Mid49 [*F*_(2,11)_ = 4.764, *p* = 0.039], Holm-sidak was used for post hoc multiple comparisons. **O** GTPase activity of Drp1. Data are presented as mean ± SEM; *p < 0.05, **p < 0.01.

Since Drp1 forms oligomers on mitochondria, we assessed Drp1 oligomers using a cross-linking method that detects both oligomeric and monomeric Drp1. In the mitochondrial fraction of sdy hippocampi, there were more monomeric Drp1 and fewer higher-order Drp1 oligomers than that of WT hippocampi, while the whole-cell lysate of WT and sdy hippocampi had no significant difference in monomeric or oligomeric Drp1 (Fig. 4A–C). Transduction of cultured hippocampal neurons with lentivirus expressing dysbindin-1C but not -1A increased higher-order Drp1 oligomers and decreased monomeric Drp1 in the mitochondrial fraction (Fig. 4D–F). Hence, dysbindin-1C promotes Drp1 oligomerization on mitochondria.

To test whether dysbindin-1 is involved in the recruitment of Drp1 to mitochondria during activity-induced mitochondrial fission, we used optical stimulation to depolarize WT hippocampal neurons co-transfected with the YFP-Drp1, mitoDsRed, and ChR2 constructs. Neurons were illuminated and imaged before and after stimulation. Light stimulation increased the number and size of YFP-Drp1 puncta on mitochondria in control cells (Supplementary Fig. 5A–C). In neurons transfected with the dysbindin-1 siRNA plasmid, but not scrambled siRNAs, the effect of optical stimulation on Drp1 was abolished (Supplementary Fig. 5A–C). Light stimulation-induced Drp1 accumulation on mitochondria was also blocked in sdy neurons (Supplementary Fig. 5D–F). The larger and more Drp1 puncta on mitochondria in stimulated neurons are consistent with increased Drp1 oligomerization on mitochondria induced by neural depolarization with KCl (50 mM in Tyrode’s solution) (Supplementary Fig. 5G–I). Hence, dysbindin-1 is required for neural activity-induced Drp1 oligomerization on mitochondria.

How does dysbindin-1 regulate Drp1 oligomerization? Dysbindin-1 has been detected on mitochondrial outer membranes by electron microscopy (EM) [22]. It may bind to Drp1 and Drp1 receptors. To test this possibility, we first tested if dysbindin-1 is indeed localized on mitochondria. Because our dysbindin-1 antibody and the commercially available dysbindin-1 antibodies are not suitable for immunocytochemistry, we used immunoblotting to detect dysbindin-1 in the mitochondrial fraction. Dysbindin-1C was present in the mitochondrial fraction and the PSD fraction as previously reported [30] (Supplementary Fig. 6A, B). To test if dysbindin-1 on mitochondria colocalizes with Drp1, we co-transfected cultured WT hippocampal neurons (DIV14) with the mitoDsRed and dysbindin-1C-GFP plasmids and stained them with the Drp1 antibody at DIV17. Dysbindin-1C-GFP and Drp1 doubly positive structures were detected on mitochondria (Supplementary Fig. 6C).

We next tested if dysbindin-1 interacts with Drp1 by transfecting HEK-293 cells with YFP-Drp1 along with HA-tagged dysbindin-1C or -1A, and then used an anti-HA antibody to pull down dysbindin-1 2 days later. Drp1 was detected in the immunoprecipitation product from cells transfected with the dysbindin-1C but not with -1A plasmid (Fig. 4G), indicating that dysbindin-1C interacts with YFP-Drp1.

To test whether dysbindin-1C binds to endogenous Drp1, we transduced cultured hippocampal neurons with lentivirus expressing HA-dysbindin-1C or -1A and used an anti-HA antibody for immunoprecipitation. Drp1 co-precipitated with dysbindin-1C, but not with -1A (Fig. 4H). Moreover, we were able to pull down endogenous Drp1 from the lysate of hippocampal tissues and primary neurons with a Drp1 antibody (Fig. 4I, J). These results indicate that dysbindin-1C interacts with Drp1. The interaction between dysbindin-1C and Drp1 was inhibited by the N-terminal region of dysbindin-1A (Fig. 4K).

To test if dysbindin-1C interacts with mitochondrial fission factor (Mff) and mitochondrial dynamics proteins of 49 and 51 kDa (Mid49 and Mid51), the three Drp1 receptors playing the predominant role in mitochondrial fission in mammalian cells [31], we transfected HEK-293 cells with HA-tagged dysbindin-1C and used an anti-HA antibody for immunoprecipitation 3 days later. Mid49 and Mid51, but not Mff, were detected in the immunoprecipitation product (Fig. 4L), indicating that dysbindin-1C interacts with Mid49 and Mid51. Because we were not able to find suitable antibodies for immunoprecipitation of endogenous Mid49 or Mid51, we used the yeast two-hybrid interaction assay (Y2H) to confirm the interaction of dysbindin-1C with Mid49 and Mid51 and test if the interaction is direct. Y2H showed that dysbindin-1C interacts with Mid49, Mid51, and Drp1, but not Mff (Supplementary Fig. 6D), indicating that dysbindin-1C directly binds to Mid49, Mid51, and Drp1. Consistent with previous reports, Drp1 binds to Mff, Mid49, and Mid51 in Y2H (Supplementary Fig. 6D) [32–34].

To test if dysbindin-1C influences the interaction between Drp1 and its receptors on mitochondria, we transfected HEK-293 cells with the dysbindin-1C or dysbindin-1A plasmid and pulled down endogenous Drp1 with the Drp1 antibody. Dysbindin-1C increased Mid49 but not Mid50 or Mff in the immunoprecipitation product, and dysbindin-1A had no effect (Fig. 4M, N). These results suggest that dysbindin-1C promotes the interaction between Drp1 and Mid49.

To test if dysbindin-1C affects the GTPase activity of Drp1, we transfected HEK-293 cells with the dysbindin-1C or dysbindin-1A plasmid and measured the GTPase activity of Drp1 3 days later. The GTPase activity of Drp1 was comparable in all groups (Fig. 4O), indicating that dysbindin-1 does not affect the GTPase activity of Drp1.

What is the cellular signal that dysbindin-1 senses to promote Drp1 translocation to mitochondria? Since dysbindin-1 is involved in activity-induced mitochondrial fission, and neural activity increases ATP consumption to generate action potentials, membrane repolarization, and biochemical cascades [3], we tested if cellular ATP change can trigger dysbindin-1 translocation to mitochondria. To this end, we treated WT and sdy hippocampal slices with oligomycin (an inhibitor of ATP synthase; 10 μM, 30 min) to reduce cellular ATP and then prepared the total lysate and mitochondrial fraction for immunoblotting. Oligomycin increased higher-order Drp1 oligomers and decreased monomeric Drp1 in the mitochondrial fraction of WT but not sdy slices (Supplementary Fig. 6E–G). Oligomycin also increased dysbindin-1C in the mitochondrial fraction of WT slices without altering dysbindin-1C in the total lysate (Supplementary Fig. 6H, I). These results indicate that a cellular ATP drop induces dysbindin-1C translocation to mitochondria and Drp1 oligomerization on mitochondria.

It has been reported that neural depolarization promotes mitochondrial fission and that stimulations inducing spine formation increase mitochondria in or at the base of dendritic spines [2, 29]. Having found that dysbindin-1 regulates activity-induced mitochondrial fission, we tested whether it also affects mitochondria in or at the base of dendritic spines. To this end, we transfected cultured hippocampal neurons (DIV14) with mitoDsRed, Venus, and ChR2 constructs and illuminated neurons as described above. The same neuron was imaged before and after stimulation. Light stimulation doesn’t affect spine number or the proportion of dendritic spines with mitochondria in the spine or at the base of spines (Supplementary Fig. 7A–C). The lack of mitochondrial translocation to spines is likely because mitochondrial translocation to dendritic spines is associated with spine formation [2], while 40-Hz light stimulation is insufficient to induce spine formation.

Moreover, we used EM to examine the effect of neural depolarization on mitochondria distribution. Primary hippocampal neurons from WT and sdy mice were transduced with AAV expressing ChR2 and stimulated with light pulses (473 nm, 40 Hz, 1 ms pulse duration, 2 mW/mm^2^, 10 min) before fixation for EM. Mitochondria were found near the postsynaptic density (PSD) in both WT and sdy neurons (Supplementary Fig. 7D). Light stimulation increased the number of mitochondria within 500 nm of the PSD in WT neurons (Supplementary Fig. 7E). This effect, however, was not observed in sdy neurons, even though the total number of mitochondria was comparable in sdy and WT neurons (Supplementary Fig. 7F). These results indicate that dysbindin-1 is involved in the mitochondrion increase near synapses induced by 40-Hz stimulation.

Taken together, these findings indicate that dysbindin-1C binds to Drp1 and Drp1 receptors and promotes Drp1 oligomerization on mitochondria.

### Gamma oscillations are impaired in sdy mice

**Fig. 5:**
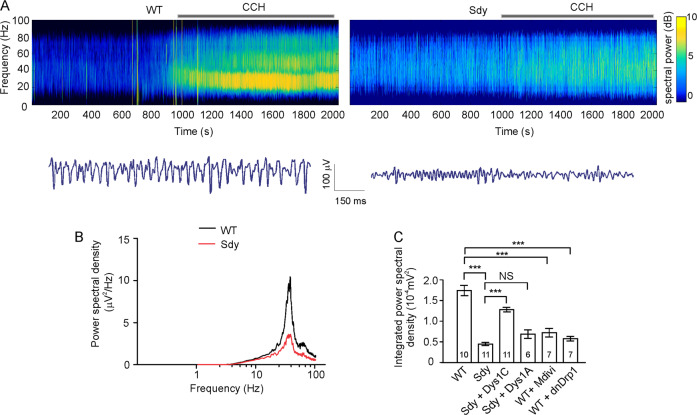
Gamma oscillations are impaired in the hippocampal CA3 region of sdy slices. Hippocampal slices were prepared from sdy mice and their WT littermates for recordings in the CA3 area. **A** Sample power spectrogram (top) and voltage traces after CCH treatment (bottom); 20 μM CCH was added to the bath at 10 min after baseline recordings. **B** Mean power spectral density of LFPs. **C** Integrated power spectral density of LFPs at 20–80 Hz recorded from the CA3 region from WT mice and their sdy littermates injected with designated lentivirus in the CA3 region; 20 μM CCH was added to the bath at 10 min after baseline recording; slices treated with Mdivi-1 were perfused with 100 μM Mdivi-1 at 10 min before recording and throughout the experimental period; one-way ANOVA was used to compare across groups [*F*_(5, 46)_ = 39.980, *p* = 0.001]; Mann–Whitney *U* test was used to compare WT vs. sdy and sdy vs. Dys1A; Student’s *t*-test was used to compare sdy vs. Dys1C, WT vs. Mdivi-1 and WT vs. dnDrp1; n in the bars indicates the number of brain slices from four animals. Data are presented as mean ± SEM; ***p < 0.001.

Given the great energy expenditure of postsynaptic membrane repolarization, the recruitment of mitochondria to postsynaptic sites following neural activation is predicted to be beneficial to high-frequency neural activities. Attenuation of this process in dysbindin-1 deficient cells, therefore, may weaken the ability of neurons to undergo such high-frequency activities as gamma oscillations. We tested this possibility by examining gamma oscillations. WT hippocampal slices (6–8 weeks of age) were treated with the cholinergic agonist carbachol (CCH, 20 μM), which induces CA3-dependent gamma oscillations [11], and recorded for LFPs in the CA3 area. Because CCH treatment induced a hump at 20–80 Hz in the power spectrum of LFPs, we measured the integrated power of LFPs in this frequency range and found that it was decreased in sdy slices (Fig. 5). To test which dysbindin-1 isoform is involved, we injected lentivirus expressing dysbindin-1C or -1A driven by the CaMKIIα promoter into the CA3 area of sdy mice (5 weeks of age) and prepared hippocampal slices 2 weeks later. The C, but not the A, isoform of dysbindin-1 largely restored gamma oscillations in sdy slices (Fig. 5C). Hence, dysbindin-1C, but not -1A, is required for gamma oscillations.

### Defective mitochondrial fission underlies the gamma-oscillation reduction in sdy mice

The findings of mitochondrial-fission and gamma-oscillation deficits in sdy mice prompted us to test whether mitochondrial fission is involved in gamma oscillations. We inhibited mitochondrial fission with the Drp1 inhibitor Mdivi-1 or by transducing CA3 neurons with lentivirus expressing a dominant-negative mutant of Drp1 (dnDrp1; Drp1-K38A; driven by the CaMKIIα promoter). Both Mdivi-1 and dnDrp1 decreased the power of gamma oscillations induced by CCH in WT hippocampal slices (Fig. 5C). Hence, mitochondrial fission is required for gamma oscillations.

**Fig. 6:**
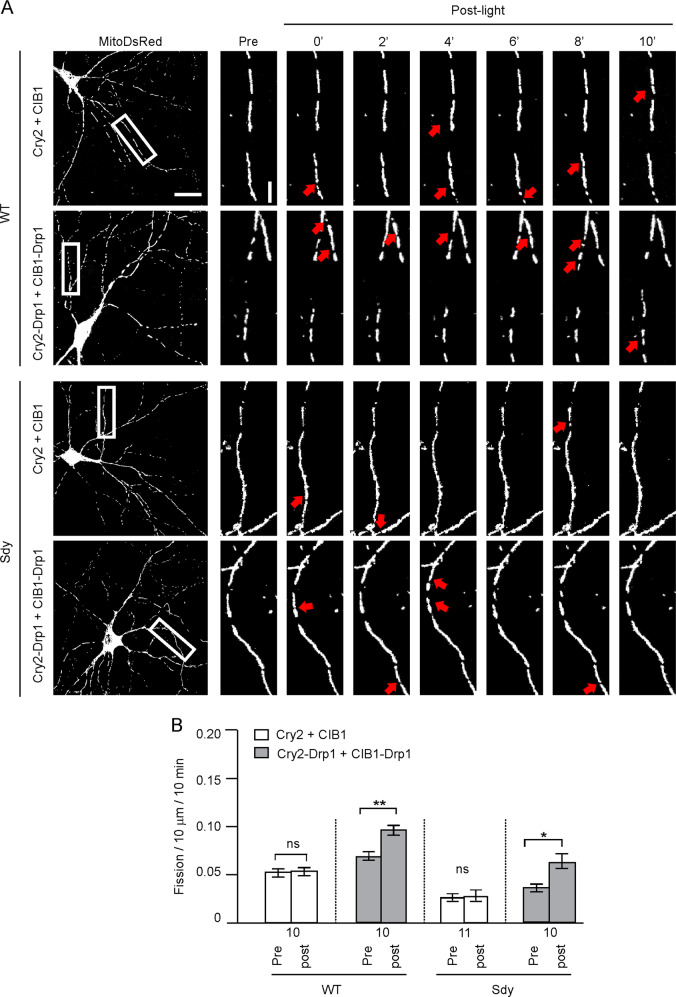
Light stimulation of Cry2-Drp1 and CIB1-Drp1 restores neural mitochondrial fission in sdy neurons. Cultured WT and sdy hippocampal neurons were transfected with mitoDsRed plasmids along with Cry2-Drp1 and CIB1-Drp1 or along with Cry2 and CIB1, and stimulated with 488-nm light for 10 min. **A** Representative images; scale bar, 20 μm for low-magnification images and 5 μm for high-magnification images. **B** Quantification for **A**; paired Student’s *t*-test was used to test for the effect of light stimulation. Data are presented as mean ± SEM; numbers under the *x*-axis indicate the number of neurons; *p < 0.05, **p < 0.01.

Next, we tested whether the mitochondrial fission deficit in sdy mice contributes to their reduction of gamma oscillations. To this end, we designed a light-inducible mitochondrial fission system by fusing Drp1 with *Arabidopsis* cryptochrome 2 (Cry2) and cryptochrome-interacting basic-helix-loop-helix 1 (CIB1). Cry2 is a FAD-binding protein that undergoes a conformational change to interact with CIB1 upon blue light illumination [35]. We generated two constructs, one expressing a Drp1 and Cry2 fusion protein and the other one expressing a Drp1 and CIB1 fusion protein. We predicted that light stimulation of these fusion proteins will induce Cry2 and CIB1 interaction, thereby promoting the oligomerization of Drp1 in the fusion proteins and subsequent mitochondrial fission. To test this prediction, we transfected cultured hippocampal neurons (DIV14) with the Cry2-Drp1 and CIB1-Drp1 constructs and illuminated them with blue light (488 nm, 1 mW/mm^2^, 10 min) at 3 days after transfection. Transfected neurons were imaged before and after illumination. Light stimulation increased colocalization between Cry2-Drp1 and CIB1-Drp1 (Supplementary Fig. 8) and mitochondrial fission (Fig. 6). By contrast, in cells transfected with the Cry2 and CIB1 plasmids, although illumination increased the size of CIB1 puncta and the colocalization between Cry2 and CIB1, mitochondrial fission was unchanged (Supplementary Fig. 8 and Fig. 6). Light stimulation of Cry2-Drp1 and CIB1-Drp1 restored mitochondrial fission in sdy neurons (Fig. 6) These results indicate that the Cry2-Drp1 and CIB1-Drp1 system can be used to enhance Drp1 oligomerization.

**Fig. 7:**
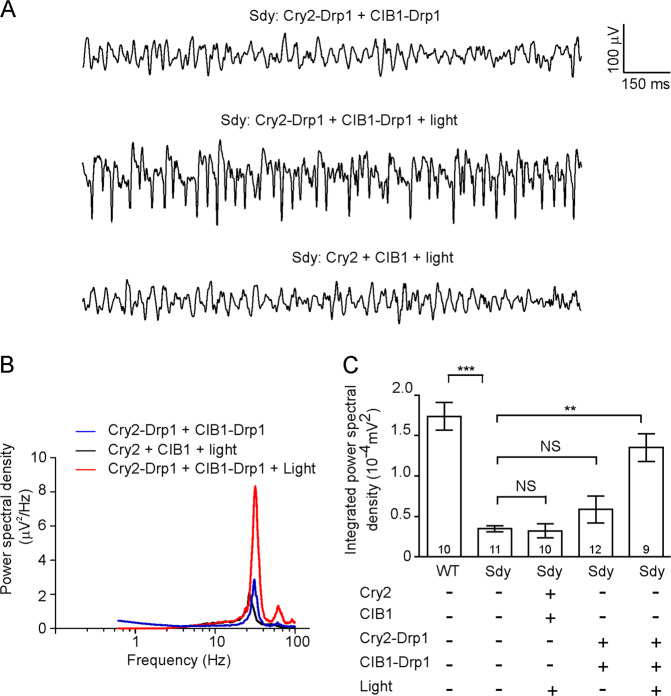
Light-induced Drp1 oligomerization restores gamma oscillations in sdy slices. Hippocampal slices were prepared from sdy mice injected with designated lentivirus and recorded in the CA3 region for LFPs; gamma oscillations were induced by perfusion with 20 μM CCH. **A** Sample traces of local field potentials recorded without (top) or at 10 min after (middle, bottom) light stimulation. **B** Mean power spectral density of LFPs. **C** Integrated power spectral density of LFPs at 20–80 Hz. One-way ANOVA on ranks (*H* = 40.038, DF = 4, *p* = 0.001) was used to compare across groups; Student’s *t*-test was used to compare unstimulated and light-stimulated sdy mice transduced with Cry2 and CIB1 virus; Mann–Whitney *U* test was used to compare unstimulated and light-stimulated sdy slices transduced with Cry2-Drp1 and CIB1-Drp1 viruses, and sdy vs. WT; n in the bars indicates the number of brain slices from four animals. Data are presented as mean ± SEM; **p < 0.01; ***p < 0.001.

To test the effect of light-induced mitochondrial fission on gamma oscillations, we injected lentivirus expressing Cry2-Drp1 and CIB1-Drp1 driven by the CaMKIIα promoter into the CA3 region of sdy mice (5–6 weeks of age) and assessed CCH-induced gamma oscillations in hippocampal slices 2 weeks later. Light stimulation largely restored gamma oscillations in sdy slices transduced with the Cry2-Drp1 and CIB1-Drp1 virus, but not with the Cry2 and CIB1 virus (Fig. 7). In addition, CCH increased dysbindin-1 translocation to mitochondria and Drp1 oligomerization on mitochondria, and the latter process was inhibited in sdy mice (Supplementary Fig. 9). These results indicate that the impairment of gamma oscillations in sdy mice is attributable to defective mitochondrial fission.

Mitochondrial fission is associated with changes in mitochondrial membrane potential (MMP) which is coupled to the generation of such mitochondrial products as ATP and reactive oxygen species (ROS) [36–39]. To test whether MMP is also altered in dysbindin-1 deficient neurons, we stained WT and sdy hippocampal slices with tetramethylrhodamine ethyl ester (TMRE), a fluorescent dye taken up by mitochondria proportionally to MMP. Sdy slices accumulated less TMRE (Supplementary Fig. 10A, B), indicating that they have depolarized MMP.

We assessed ROS with MitoSOX Red (a mitochondrion-targeted dye that emits red fluorescence after being oxidized by superoxide), and cellular ATP from phosphorylated AMP-activated protein kinase (p-AMPK), which is elevated when the ATP/AMP and ATP/ADP ratios decrease. Both p-AMPK and MitoSOX fluorescence increased in sdy hippocampal cells (Supplementary Fig. 10C–F). Hence, cellular ATP decreased, while ROS increased in dysbindin-1 deficient cells.

To test whether the fission defect contributes to the MMP alteration in sdy mice, we enhanced fission in sdy hippocampal neurons using the Cry2-Drp1 and CIB1-Drp1 system. Increasing mitochondrial fission by light stimulation doesn’t affect TMRE uptake in sdy neurons (Supplementary Fig. 11A, B). Likewise, mitoSOX fluorescence intensity in sdy neurons was unchanged by light stimulation (Supplementary Fig. 11A, C). These results indicate that MMP depolarization and ROS increase in sdy neurons are not caused by defective mitochondrial fission.

In WT neurons stimulated with light, TMRE intensity in dendritic spines increased, while mitoSOX signals remained unchanged (Supplementary Fig. 11A, D, E). These findings suggest that mitochondrial fission facilitates the enrichment of mitochondria with hyperpolarized MMP in postsynaptic sites. It has been reported that 85% of mitochondrial fission events result in one daughter mitochondria with hyperpolarized MMP and one with depolarized MMP, and the two daughter mitochondria move apart to inhabit new subcellular compartments [37, 40]. It is perceivable that after fission, the daughter mitochondria with hyperpolarized MMP are more likely to move to postsynaptic sites with high energy demands.

Taken together, these findings indicate that mitochondrial fission is required for gamma oscillations and that the attenuation of gamma oscillations in sdy mice is attributable to defective mitochondrial fission.

### Light-induced mitochondrial fission restores in vivo gamma oscillations and NOR in sdy mice

Sdy mice have deficits in gamma oscillations and behaviors associated with gamma oscillations, such as NOR [41, 42]. The NOR test is based on the animal’s natural tendency to spend more time exploring novel than familiar objects. It assesses visual learning and memory, one of the key cognitive domains impaired in schizophrenia and is a widely used preclinical cognitive assay [43–47].

**Fig. 8:**
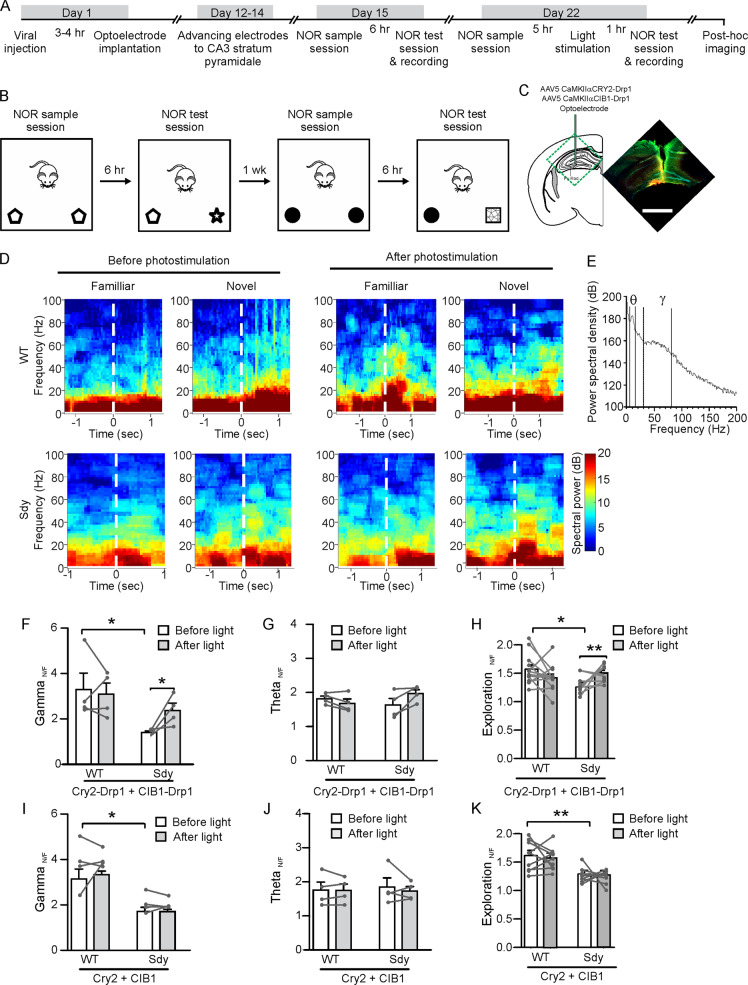
Light-induced mitochondrial fission rescues in vivo gamma oscillations and novel object recognition (NOR) in sdy mice. 5-week-old sdy mice and their WT littermates were injected with virus and implanted with optoelectrodes or optic fibers in the CA3 region, and then subjected to in vivo recording and the NOR test. **A** Experimental schedule. **B** NOR test protocol. **C** The site of viral injection and placement of optoelectrode. The image shows the expression of Cry2-Drp1 (red), CIB1-Drp1 (green), and DAPI staining (Cyan) at the injection site; scale bar, 1 mm. **D** The histogram shows the power spectral density (PSD) of LFPs vs. frequencies. The four inflection points at 6, 15, 30, 80 Hz as indicated by the dash lines were used to define the gamma and the theta range. **E** Time-frequency spectrogram of LFP oscillations before and after object exploration during the test session of NOR in mice injected with Cry2-Drp1 and CIB1-Drp1 viruses. The dashed line indicates the time when the mouse started the exploration. **F**, **G** Ratio of the gamma-range integrated power spectral density during novel object exploration to that during familiar object exploration (Gamma _N/F_) before and after light stimulation. **H**, **I** Ratio of the theta range integrated power spectral density during novel object exploration to that during familiar object exploration (Theta _N/F_) before and after light stimulation. **J**, **K** Ratio of time exploring the novel object to that exploring the familiar object (Exploration _N/F_) before and after light stimulation. Student’s *t*-test was used to compare sdy vs. WT and paired Student’s *t*-test was used to compare before vs. after light stimulation. Data are presented as mean ± SEM; n in the bars indicates the number of mice; *p < 0.05, **p < 0.01. (color figure online).

Having found that light-induced mitochondrial fission restores carbachol-induced gamma oscillations in sdy mice, we proceeded to test whether it influences sdy mice’s in vivo gamma oscillations and NOR. We introduced the light-inducible mitochondrial fission system into the CA3 region of sdy mice and their WT littermates via viral injection. At 3–4 h after viral injection, a microdrive-controlled optoelectrode consisting of 8 tetrodes and an optic fiber was implanted into the CA3 region. After surgery, the animals were subjected to two NOR tests using the no habituation, 6 h intersession interval protocol as described previously, because animals exhibit more active explorations of novel objects with this protocol [48]. LFPs were recorded during the test session. Different objects were used as familiar and novel objects in the first and the second NOR test. The first NOR test did not involve light stimulation and was conducted 2 weeks after surgery. The second NOR test, conducted 3 weeks after surgery, included light stimulation (473 nm, 10 min, 2 mW/mm^2^) applied through the optical fiber 1 h before the test session (Fig. 8A–C).

We analyzed LFP oscillations recorded during the first session of exploring the familiar and the novel object in the test session. When WT mice explored an object, the LFP power spectrum exhibited a gamma-band hump at 30–80 Hz and a theta-band hump at 6–15 Hz (Fig. 8D, E), we, therefore, analyzed the integrated oscillation power in these frequency ranges. Since both gamma and theta oscillations are altered during running [49, 50], to reduce the effect of inter-individual variability of locomotor activities on the analysis of LFP oscillations, we normalized the power of LFP oscillations during object exploration to that during the pre-exploration period.

In the first NOR test, the ratio of the gamma-oscillation power during novel object exploration to that during familiar object exploration (Gamma _N/F_) was ~3.2 in WT mice (Fig. 8D, F), indicating that novel objects elicit more powerful gamma oscillations than familiar objects. This is consistent with the previous finding [42]. The increase in gamma oscillations associated with novel object exploration was comparable in mice expressing Cry2-Drp1 and CIB1-Drp1 and in those expressing Cry2 and CIB1 (Fig. 8D, F, G), indicating that Drp1 overexpression had no effect on this phenomenon. The Gamma _N/F_ ratio in sdy mice, however, was only ~1.55 (Fig. 8D, F). In the second NOR test, while light stimulation doesn’t affect Gamma _N/F_ in WT mice and sdy mice injected with virus expressing CIB1 and Cry2, it increased Gamma _N/F_ in sdy mice injected with the CIB1-Drp1 and the Cry2-Drp1 virus (Fig. 8D, F, G). These results indicate that the mitochondrial fission deficit in sdy mice contributes to their reduced in vivo gamma oscillations. Unlike Gamma _N/F_, the ratio of novel to familiar objects for theta oscillations (Theta _N/F_) was comparable in WT and sdy mice and was left intact after light stimulation (Fig. 8D, H, I), suggesting that dysbindin-1 and mitochondrial fission predominantly influence high-frequency LFP oscillations.

To test for the effect of light-induced mitochondrial fission on NOR, WT and sdy mice were injected with the Cry2-Drp1 and CIB1-Drp1 virus or control (Cry2 and CIB1) virus followed by implantation with an optic fiber over the CA3 region, and then went through two NOR tests, the second of which included a light stimulation as described above. The ratio of time spent exploring the novel object to that spent exploring the familiar object (Exploration _N/F_) was calculated for the first period during which the total duration of object (both familiar and novel) exploration reached 15 s. In the first NOR test, sdy mice explored the novel object less than WT mice as indicated by a lower Exploration _N/F_ (Fig. 8J, K). At 1 h before the test session of the second NOR test, mice were stimulated through the implanted optic fiber at 473 nm for 10 min (2 mW/mm^2^). While photostimulation doesn’t affect Exploration _N/F_ in either sdy or WT mice injected with the control virus, it increased Exploration _N/F_ in sdy mice expressing Cry2-Drp1 and CIB1-Drp1 (Fig. 8J, K). Exploration _N/F_ in WT mice expressing Cry2-Drp1 and CIB1-Drp1 was not significantly altered by light stimulation (Fig. 8J). Hence, the NOR deficit in sdy mice can be rescued by enhancing mitochondrial fission.

Taken together, these findings indicate that a decrease in mitochondrial fission causes impairments of in vivo gamma oscillations and NOR.

## Discussion

Although several neural network models have been proposed to explain the generation of neural synchronization during gamma oscillations [9], the cellular mechanisms by which the high, local energy demand at synapses is met during gamma oscillations remain largely unclear. This study discovers the role of mitochondrial fission in gamma oscillations and an unexpected function of the schizophrenia-associated protein dysbindin-1 in regulating mitochondrial fission.

We found that mitochondria are elongated and mitochondrial fission is reduced in sdy mice. This is attributable to the loss of dysbindin-1C. Hence, dysbindin-1 does not only control the subcellular localization of proteins, synaptic release, and synaptic plasticity as previously reported [16–21] but also plays a role in the regulation of mitochondrial fission. We have several lines of evidence supporting that dysbindin-1 regulates mitochondrial fission through Drp1. The immunoprecipitation and Y2H assays show that dysbindin-1C interacts with the mitochondrial fission protein Drp1 and its adaptors Mid49 and Mid51. Our analyses of Drp1 oligomers and Drp1 puncta on mitochondria indicate that dysbindin-1C promotes Drp1 oligomerization on mitochondria. In sdy mice, higher-order Drp1 oligomers on mitochondria are reduced, while dysbindin-1C overexpression in neurons increases higher-order Drp1 oligomers on mitochondria. In dysbindin-1 deficient neurons, the increase in Drp1 puncta on mitochondria associated with neuronal depolarization-induced mitochondrial fission is abolished. The N-terminal region of dysbindin-1A is missing in dysbindin-1C and appears to be responsible for the functional difference between dysbindin-1C and dysbindin-1A in mitochondrial fission as it inhibits the interaction between dysbindin-1C and Drp1.

Consistent with earlier reports that neural activity promotes mitochondrial fission [2, 29], we found that optical stimulation in the gamma band increases mitochondrial fission. After fission, mitochondria break apart to inhabit new subcellular compartments and more mitochondria are found in the vicinity of postsynaptic sites. In dysbindin-1 deficient neurons, activity-induced mitochondrial fission is impaired and 40-Hz optical stimulation no longer increases mitochondria near postsynaptic sites. Hence, dysbindin-1 is involved in activity-dependent redistribution of mitochondria to the dendritic area near postsynaptic sites. However, 40-Hz optical stimulation does not alter the abundance of mitochondria in dendritic spines. This is in contrast to 100-Hz tetanic electrical stimulation which increases mitochondria in dendritic spines [2]. 40-Hz optical stimulation and 100-Hz tetanic electrical stimulation are different in such features as frequency, pulse width, and intensity. These differences could underlie their distinct effects on dendritic spines and mitochondria. Indeed, while 40-Hz optical stimulation doesn’t affect spine number, 100-Hz tetanic electrical stimulation induces spine formation and infiltration of mitochondria into newly formed spines [2].

Earlier EM studies show that a small amount of dysbindin-1 proteins is present on mitochondrial outer membranes and that dysbindin-1C in synapses is predominantly associated with postsynaptic densities [22, 30]. Consistent with these reports, we detected dysbindin-1C in the mitochondrial and the PSD fractions. In hippocampal cells treated with CCH, which induces gamma oscillations, or depolarized with high K^+^, dysbindin-1C on mitochondria increases. These findings indicate that neural activity induces dysbindin-1C translocation to mitochondria where it binds to Drp1 and its adaptor proteins to promote Drp1 oligomerization and subsequent mitochondrial fission and redistribution to postsynaptic sites.

Because neural repolarization is an energy-demanding process during synaptic transmission [3], mitochondria are important for synaptic activities, especially those at high-frequencies like gamma oscillations. This notion is supported by the tight correlation between the power of gamma oscillations and the hemodynamic response in the cortex and oxygen consumption in the hippocampus, and the dependence of hippocampal gamma oscillations on strong mitochondrial function [7, 8, 51]. The ATP demand at the postsynaptic site is believed to greatly increase during gamma oscillations. Little is known, however, about how mitochondria accommodate this change. It has been reported that 85% of mitochondrial fission events result in one daughter mitochondria with hyperpolarized transmembrane potential (more metabolically active) and one with depolarized transmembrane potential (less metabolically active), and the two daughter mitochondria move apart to inhabit new subcellular compartments [37, 40]. We found that TMRE uptake in dendritic spines increased after light-induced mitochondrial fission, indicating that mitochondrial fission facilitates the enrichment of mitochondria with higher activities in postsynaptic sites. Hence, mitochondrial fission promotes the dispatch of mitochondria to postsynaptic sites to meet the rapid increase in ATP demand near synapses during gamma oscillations. This newly identified function of mitochondrial fission in neural synchronization is beyond its recognized roles in controlling mitochondrial morphology, mitochondrial number, mitophagy, and apoptosis [52, 53].

To test the impact of deficient mitochondrial fission on gamma oscillations, we developed a light-inducible mitochondrial fission system. We examined both CCH-induced ex vivo and novel object exploration-associated, in vivo gamma oscillations. Light-induced mitochondrial fission largely, but not fully, restored gamma oscillations in sdy mice. The incomplete rescue may be because only a portion of CA3 neurons was transduced by virus. It is also possibly due to the reduction of PV neurons and inhibitory synaptic inputs to excitatory neurons which can be attributable, at least in part, to decreased BDNF exocytosis from excitatory neurons and reduced excitatory drive to GABAergic neurons [18, 30]. The role of inhibitory dysfunction in sdy mice in their gamma oscillation phenotype is beyond the scope of this study and warranting future studies to address.

Our findings do not exclude the possibility that multiple mechanisms contribute to impaired gamma oscillations in sdy mice. Nevertheless, the enhancement of both CCH-induced and in vivo gamma oscillations by light-induced mitochondrial fission indicates that less mitochondrial fission has deleterious effects on gamma oscillations.

The mRNA and protein expression of dysbindin-1 decrease in the brains of people with schizophrenia in regions commonly affected by schizophrenia including the prefrontal cortex and hippocampus [16]. Our results suggest that a decrease in dysbindin-1 expression can lead to aberrant gamma oscillations, which have been implicated in cognitive impairment. For example, the power of gamma oscillations increases during tests for complex cognitive processing and correlates positively with the subject’s performance [9, 54–58]. This increase, however, is absent in people with schizophrenia, and this deficit correlates with their poor cognitive performance [54, 57, 59–61]. The improvements of gamma oscillations and NOR by light-induced mitochondrial fission suggest that facilitating mitochondrial fission may ameliorate cognitive dysfunction. Although mitochondrial fission has not been previously linked to psychiatric disorders, anomalous mitochondrial morphology and number have been noted in the brains of individuals with schizophrenia [62], and they may arise from aberrant mitochondrial fission.

In sum, our study sheds new light on the physiological function of mitochondrial fission and cellular mechanisms of gamma oscillations.

## Supplementary information

Supplementary Materials, Methods, Figure legends

Supplemental Figure 1

Supplemental Figure 2

Supplemental Figure 3

Supplemental Figure 4

Supplemental Figure 5

Supplemental Figure 6

Supplemental Figure 7

Supplemental Figure 8

Supplemental Figure 9

Supplemental Figure 10

Supplemental Figure 11

## References

[1] van der Bliek AM, Shen Q, Kawajiri S (2013). Mechanisms of mitochondrial fission and fusion. Cold Spring Harb Perspect Biol.

[2] Li Z, Okamoto K, Hayashi Y, Sheng M (2004). The importance of dendritic mitochondria in the morphogenesis and plasticity of spines and synapses. Cell.

[3] Harris JJ, Jolivet R, Attwell D (2012). Synaptic energy use and supply. Neuron.

[4] Rolfe DF, Brown GC (1997). Cellular energy utilization and molecular origin of standard metabolic rate in mammals. Physiological Rev.

[5] Rangaraju V, Calloway N, Ryan TA (2014). Activity-driven local ATP synthesis is required for synaptic function. Cell.

[6] Pathak D, Shields LY, Mendelsohn BA, Haddad D, Lin W, Gerencser AA (2015). The role of mitochondrially derived ATP in synaptic vesicle recycling. J Biol Chem.

[7] Huchzermeyer C, Albus K, Gabriel HJ, Otahal J, Taubenberger N, Heinemann U (2008). Gamma oscillations and spontaneous network activity in the hippocampus are highly sensitive to decreases in pO2 and concomitant changes in mitochondrial redox state. J Neurosci.

[8] Kann O, Huchzermeyer C, Kovacs R, Wirtz S, Schuelke M (2011). Gamma oscillations in the hippocampus require high complex I gene expression and strong functional performance of mitochondria. Brain.

[9] Buzsaki G, Wang XJ (2012). Mechanisms of gamma oscillations. Annu Rev Neurosci.

[10] Fries P (2009). Neuronal gamma-band synchronization as a fundamental process in cortical computation. Annu Rev Neurosci.

[11] Fisahn A, Pike FG, Buhl EH, Paulsen O (1998). Cholinergic induction of network oscillations at 40 Hz in the hippocampus in vitro. Nature.

[12] Mann EO, Suckling JM, Hajos N, Greenfield SA, Paulsen O (2005). Perisomatic feedback inhibition underlies cholinergically induced fast network oscillations in the rat hippocampus in vitro. Neuron.

[13] Traub RD, Whittington MA, Colling SB, Buzsaki G, Jefferys JG (1996). Analysis of gamma rhythms in the rat hippocampus in vitro and in vivo. J Physiol.

[14] Williams JH, Kauer JA (1997). Properties of carbachol-induced oscillatory activity in rat hippocampus. J Neurophysiol.

[15] Oren I, Hajos N, Paulsen O (2010). Identification of the current generator underlying cholinergically induced gamma frequency field potential oscillations in the hippocampal CA3 region. J Physiol.

[16] Talbot K, Ong WY, Blake DJ, Tang J, Louneva N, Carlson GC et al. Dysbindin-1 and its protein family. Handbook of Neurochemistry and Molecular Neurobiology: Schizophrenia. 3rd ed. Boston, MA: Springer. 2009. p.107–241.

[17] Schmieg N, Rocchi C, Romeo S, Maggio R, Millan MJ, Mannoury la Cour C (2016). Dysbindin-1 modifies signaling and cellular localization of recombinant, human D(3) and D(2) receptors. J Neurochem.

[18] Yuan Q, Yang F, Xiao Y, Tan S, Husain N, Ren M (2016). Regulation of brain-derived neurotrophic factor exocytosis and gamma-aminobutyric acidergic interneuron synapse by the schizophrenia susceptibility gene dysbindin-1. Biol Psychiatry.

[19] Glen WB, Horowitz B, Carlson GC, Cannon TD, Talbot K, Jentsch JD (2014). Dysbindin-1 loss compromises NMDAR-dependent synaptic plasticity and contextual fear conditioning. Hippocampus.

[20] Mullin AP, Sadanandappa MK, Ma W, Dickman DK, VijayRaghavan K, Ramaswami M (2015). Gene dosage in the dysbindin schizophrenia susceptibility network differentially affect synaptic function and plasticity. J Neurosci.

[21] Orozco IJ, Koppensteiner P, Ninan I, Arancio O (2014). The schizophrenia susceptibility gene DTNBP1 modulates AMPAR synaptic transmission and plasticity in the hippocampus of juvenile DBA/2J mice. Mol Cell Neurosci.

[22] Talbot K, Cho DS, Ong WY, Benson MA, Han LY, Kazi HA (2006). Dysbindin-1 is a synaptic and microtubular protein that binds brain snapin. Hum Mol Genet.

[23] Jia JM, Zhao J, Hu Z, Lindberg D, Li Z (2013). Age-dependent regulation of synaptic connections by dopamine D2 receptors. Nat Neurosci.

[24] Hu Z, Zhao J, Hu T, Luo Y, Zhu J, Li Z (2015). miR-501-3p mediates the activity-dependent regulation of the expression of AMPA receptor subunit GluA1. J Cell Biol.

[25] Wang H, Yuan Y, Zhang Z, Yan H, Feng Y, Li W (2014). Dysbindin-1C is required for the survival of hilar mossy cells and the maturation of adult newborn neurons in dentate gyrus. J Biol Chem.

[26] Talbot K, Eidem WL, Tinsley CL, Benson MA, Thompson EW, Smith RJ (2004). Dysbindin-1 is reduced in intrinsic, glutamatergic terminals of the hippocampal formation in schizophrenia. J Clin Investig.

[27] Jia JM, Hu Z, Nordman J, Li Z (2014). The schizophrenia susceptibility gene dysbindin regulates dendritic spine dynamics. J Neurosci.

[28] Tang J, LeGros RP, Louneva N, Yeh L, Cohen JW, Hahn CG (2009). Dysbindin-1 in dorsolateral prefrontal cortex of schizophrenia cases is reduced in an isoform-specific manner unrelated to dysbindin-1 mRNA expression. Hum Mol Genet.

[29] Divakaruni SS, Van Dyke AM, Chandra R, LeGates TA, Contreras M, Dharmasri PA (2018). Long-term potentiation requires a rapid burst of dendritic mitochondrial fission during induction. Neuron.

[30] Carlson GC, Talbot K, Halene TB, Gandal MJ, Kazi HA, Schlosser L (2011). Dysbindin-1 mutant mice implicate reduced fast-phasic inhibition as a final common disease mechanism in schizophrenia. Proc Natl Acad Sci USA.

[31] Mishra P, Chan DC (2014). Mitochondrial dynamics and inheritance during cell division, development and disease. Nat Rev Mol Cell Biol.

[32] Liu T, Yu R, Jin SB, Han L, Lendahl U, Zhao J (2013). The mitochondrial elongation factors MIEF1 and MIEF2 exert partially distinct functions in mitochondrial dynamics. Exp Cell Res.

[33] Palmer CS, Osellame LD, Laine D, Koutsopoulos OS, Frazier AE, Ryan MT (2011). MiD49 and MiD51, new components of the mitochondrial fission machinery. Embo Rep.

[34] Otera H, Wang CX, Cleland MM, Setoguchi K, Yokota S, Youle RJ (2010). Mff is an essential factor for mitochondrial recruitment of Drp1 during mitochondrial fission in mammalian cells. J Cell Biol.

[35] Liu H, Yu X, Li K, Klejnot J, Yang H, Lisiero D (2008). Photoexcited CRY2 interacts with CIB1 to regulate transcription and floral initiation in Arabidopsis. Science.

[36] Suzuki R, Hotta K, Oka K (2018). Transitional correlation between inner-membrane potential and ATP levels of neuronal mitochondria. Sci Rep.

[37] Twig G, Elorza A, Molina AJ, Mohamed H, Wikstrom JD, Walzer G (2008). Fission and selective fusion govern mitochondrial segregation and elimination by autophagy. EMBO J.

[38] Willems PHGM, Rossignol R, Dieteren CEJ, Murphy MP, Koopman WJH (2015). Redox homeostasis and mitochondrial dynamics. Cell Metab.

[39] Mitchell P (1961). Coupling of phosphorylation to electron and hydrogen transfer by a chemi-osmotic type of mechanism. Nature.

[40] Cagalinec M, Safiulina D, Liiv M, Liiv J, Choubey V, Wareski P (2013). Principles of the mitochondrial fusion and fission cycle in neurons. J Cell Sci.

[41] Talbot K (2009). The sandy (sdy) mouse: a dysbindin-1 mutant relevant to schizophrenia research. Prog Brain Res.

[42] Zheng C, Bieri KW, Hwaun E, Colgin LL (2016). Fast gamma rhythms in the hippocampus promote encoding of novel object-place pairings. eNeuro.

[43] Green MF, Nuechterlein KH, Gold JM, Barch DM, Cohen J, Essock S (2004). Approaching a consensus cognitive battery for clinical trials in schizophrenia: the NIMH-MATRICS conference to select cognitive domains and test criteria. Biol Psychiatry.

[44] Lyon L, Saksida LM, Bussey TJ (2012). Spontaneous object recognition and its relevance to schizophrenia: a review of findings from pharmacological, genetic, lesion and developmental rodent models. Psychopharmacology.

[45] Rajagopal L, Massey BW, Huang M, Oyamada Y, Meltzer HY (2014). The novel object recognition test in rodents in relation to cognitive impairment in schizophrenia. Curr Pharm Des.

[46] Young JW, Powell SB, Risbrough V, Marston HM, Geyer MA (2009). Using the MATRICS to guide development of a preclinical cognitive test battery for research in schizophrenia. Pharmacol Ther.

[47] Nuechterlein KH, Barch DM, Gold JM, Goldberg TE, Green MF, Heaton RK (2004). Identification of separable cognitive factors in schizophrenia. Schizophrenia Res.

[48] Leger M, Quiedeville A, Bouet V, Haelewyn B, Boulouard M, Schumann-Bard P (2013). Object recognition test in mice. Nat Protoc.

[49] Ahmed OJ, Mehta MR (2012). Running speed alters the frequency of hippocampal gamma oscillations. J Neurosci.

[50] Bender F, Gorbati M, Cadavieco MC, Denisova N, Gao X, Holman C (2015). Theta oscillations regulate the speed of locomotion via a hippocampus to lateral septum pathway. Nat Commun.

[51] Niessing J, Ebisch B, Schmidt KE, Niessing M, Singer W, Galuske RA (2005). Hemodynamic signals correlate tightly with synchronized gamma oscillations. Science.

[52] Youle RJ, van der Bliek AM (2012). Mitochondrial fission, fusion, and stress. Science.

[53] Chan DC (2012). Fusion and fission: interlinked processes critical for mitochondrial health. Annu Rev Genet.

[54] Basar-Eroglu C, Brand A, Hildebrandt H, Karolina Kedzior K, Mathes B, Schmiedt C (2007). Working memory related gamma oscillations in schizophrenia patients. Int J Psychophysiol.

[55] Howard MW, Rizzuto DS, Caplan JB, Madsen JR, Lisman J, Aschenbrenner-Scheibe R (2003). Gamma oscillations correlate with working memory load in humans. Cereb Cortex.

[56] Meltzer JA, Zaveri HP, Goncharova II, Distasio MM, Papademetris X, Spencer SS (2008). Effects of working memory load on oscillatory power in human intracranial EEG. Cereb Cortex.

[57] Cho RY, Konecky RO, Carter CS (2006). Impairments in frontal cortical gamma synchrony and cognitive control in schizophrenia. Proc Natl Acad Sci USA.

[58] Spellman TJ, Gordon JA (2015). Synchrony in schizophrenia: a window into circuit-level pathophysiology. Curr Opin Neurobiol.

[59] Kissler J, Muller MM, Fehr T, Rockstroh B, Elbert T (2000). MEG gamma band activity in schizophrenia patients and healthy subjects in a mental arithmetic task and at rest. Clin Neurophysiol.

[60] Woo TU, Spencer K, McCarley RW (2010). Gamma oscillation deficits and the onset and early progression of schizophrenia. Harv Rev Psychiatry.

[61] Gonzalez-Burgos G, Cho RY, Lewis DA (2015). Alterations in cortical network oscillations and parvalbumin neurons in schizophrenia. Biol Psychiatry.

[62] Hjelm BE, Rollins B, Mamdani F, Lauterborn JC, Kirov G, Lynch G (2015). Evidence of mitochondrial dysfunction within the complex genetic etiology of schizophrenia. Mol Neuropsychiatry.

