# Supplementary material for: Dysbindin-1 regulates mitochondrial fission and gamma oscillations
Source: Mol Psychiatry. 2021 Feb 15;26(9):4633–51. doi: 10.1038/s41380-021-01038-9 (PMC8364574; doi:10.1038/s41380-021-01038-9)
Supplement: Supplementary file 1 — Supplementary Materials, Methods, Figure legends [file 41380_2021_1038_MOESM1_ESM.pdf]

Supplementary Information

Supplementary Materials and Methods

Animals. Animals used for behavioral and *in vivo* electrophysiological tests were kept under a reversed light/dark cycle (dark: 9 am–9 pm; light: 9 pm–9 am) with access to food and water ad libitum. Each cage had 4–5 animals without surgery or only one animal with implantation. Behavioral and *in vivo* electrophysiological experiments were conducted during the dark cycle of the animal. 5-week old mice were injected with virus and tested for behaviors at 8-9 weeks of age.

Key reagents and software

REAGENT or RESOURCE	SOURCE	IDENTIFIER
**Antibodies**		
Mouse monoclonal anti-DLP1	BD Bioscience	611113
Rabbit monoclonal -anti DRP1 (D6C7)	Cell signaling	8570
Mouse monoclonal anti-HA	BioLegend	901502
Mouse IgG	Invitrogen	10400c
Rabbit polyclonal anti-Tom20	Santa Cruz	Sc-11415
Rabbit polyclonal anti-GFP	MBL	598
Rabbit polyclonal anti-HA	Santa Cruz	Sc-805
Rabbit IgG	Invitrogen	10500c

Rabbit polyclonal anti-SMCR7/MID49 Antibody	Proteintech	16413-1-AP
Rabbit polyclonal anti-MIEF1(Mid51)	Abcam	ab89944
Rabbit polyclonal anti-MFF	Cell signaling	86668
MAP2	Sigma-Aldrich	M9942
Tau	GeneTex	GTX49353
Rabbit polyclonal anti-Tom20	Proteintech	11802-1-AP
anti-dysbindin-1 antibody	a gift from Dr. Wei Li (Institute of Genetics and Developmental Biology, Chinese Academy of Sciences)	
**Chemicals**		
Carbachol	Tocris	2810
Oligomycin A	Santa Cruz	CAS 579-13-5
Mdivi-1	Sigma	M0199
DSP	Sigma	3669
SD/-His/-Leu/-Trp with Agar	Takara	630319
SD/-Leu/-Trp with Agar	Takara	630317
YPDA Medium	Takara	630464
YPDA with Agar	Takara	630307
**Critical Commercial Assays**		
Phusion Site-directed Mutagenesis kit	Thermo Scientific	F541

Yeast transformation kit	Takara	630439
GTPase Activity Kit (Colorimetric)	Novus Biologicals	602-0120
**Software and Algorithms**		
Sigmaplot	Systat Software	https://systatsoftware.com/products/sigmaplot/
Matlab	Mathworks	https://www.mathworks.com
Chronux toolbox	Chronux	http://chronux.org/
ImageJ	Fiji	https://fiji.sc/
MetaMorph	Molecular Devices	https://www.moleculardevices.com/systems/metamorph-research-imaging
Neurolucida 360	MBF Bioscience	https://www.mbfbioscience.com/neurolucida360

Plasmids. The following plasmids were purchased from Addgene: pEYFP-C1-Drp1 (#45160), FCK-ChR2-GFP (#15814), pFCK(1.3)GW (#27230), pCIBN(deltaNLS)-pmGFP (#26867), pCMV-Cry2-mCherry (#58368), pcDNA3.1(-) MID51 4xMyc Hisx6 (#44598), pcDNA3.1(-) MID49 4xMyc Hisx6 (#44596), pcDNA3 Flag MFF iso4 WT (#73590). pBHA-Drp1(1-736), pGAD-Drp1(1-736), pBHA and pGAD plasmids are gifts from Dr. Craig Blackstone (National Institute of Neurological Disorders and Stroke, National Institutes of Health). The following constructs were generated as described previously [1-3]: GW1-mitoDsRed, GW1-Venus, GW1-Dys1Amut, GW1-HA-Dys1A, pSuper-DysSiRNA, pSuper-DysSiRNAScramble. The YFP-dnDrp1 plasmid was generated by mutating the pEYFP-C1-Drp1 construct using the Phusion Site-directed Mutagenesis kit (Thermo Fisher Scientific) with the following primers: GAACGCAGAGCAGCGGAGCGAGCTCAGTGCTAGAAA and

TTTCTAGCACTGAGCTCGCTCCGCTGCTCTGCGTTC. Other plasmids were generated by cloning PCR fragments into vectors as listed in the following table:

**Plasmid**	**Vector**	**PCR template**	**Forward primer and restriction site**	**Reverse primer and restriction site**
GW1-HA-Dys1C	GW1	GW1-Dys1A	KpnI: GGGGTACCTCATGCTGTCTGCCCACTGGGAGAAG	SalI: ACGCGTCGACTTAAATGTCCTGAGTTGAGTCAC
Dys1Cmut	GW1	GW1-Dys1Amut	KpnI: GGGGTACCTCATGCTGTCTGCCCACTGGGAGAAG	SalI: ACGCGTCGACTTAAATGTCCTGAGTTGAGTCAC
ChR2	GW1	FCK-ChR2-GFP	SalI: ACGCGTCGACGCCACCATGGACTATGGCGGCGCTTTGTCT	EcoR1: CCGGAATTCTTATGGCACGGCTCCGGCCTCGGCTT

Lenti-MitoDsRed	pFCK(1.3)GW	GW1-MitoDsRed	BamHI: CGGGATCCGCCACCATGTCCGTCCTGACGCCG	EcoR1: CCGGAATTCTTACAGGAACAGGTGGTGGCG
Lenti-HA-Dys1C	pFCK(1.3)GW	GW1-Dys1C	BamHI: CGGGATCCATGTACCCATACGACGTCCCAGAC;	EcoR1: CCGGAATTCTTAAATGTCCTGAGTTGAGTCAC
Lenti-HA-Dys1A	pFCK(1.3)GW	GW1-Dys1A	BamHI: CGGGATCCATGTACCCATACGACGTCCCAGAC	EcoR1: CCGGAATTCTTAAATGTCCTGAGTTGAGTCAC
Lenti-dnDrp-1	pFCK(1.3)GW	GW1-dnDrp-YFP	BamHI: CGGGATCCGCCACCATGAGCAAGGGCGAGGAACTGT	EcoR1: CCGGAATTCGCCACCTCACCAAAGATGAGTCTCCCGG
CIB1-GFP	pCIBN(deltaNLS)-pmGFP	pCIBN(deltaNLS)-pmGFP	NheI: CTAGCTAGCGCCACCATGAATGGAGCTATAGGAG	XhoI: CCGCTCGAGCTTATACAGCTCGTCCATGCCGAGAG

CIB1-GFP-Drp1	CIB1-GFP	GW1-YFP-Drp1	XhoI: CCGCTCGAGATGGAGGCGCTAATTCCTGTCATAAAC	EcoRI: CGGAATTCTCACCAAAGATGAGTCTCCCGGATTTC
Cry2-mCherry-Drp1	pCMV-Cry2-mCherry	GW1-YFP-Drp1	XhoI: CCGCTCGAGATGGAGGCGCTAATTCCTGTCATAAAC	EcoRI: CGGAATTCTCACCAAAGATGAGTCTCCCGGATTTC
Lenti-CIB1-GFP	pFCK(1.3)GW	CIB1-GFP	BamHI: CGGGATCCGCAGAGCTGGTTTAGTGAACCGTCAG	EcoR1: CGGAATTCTTACTCGTCCATGCCGAGAGTGATC
Lenti-Cry2-mCherry	pFCK(1.3)GW	Cry2-mCherry	BamHI: CGGGATCCGCAGAGCTGGTTTAGTGAACCGTCAG	ECoR1: CGGAATTCTTACTTGTACAGCTCGTCCATGCC

Lenti-CIB1-GFP-Drp1	pFCK(1.3)GW	CIB1-GFP-Drp1	BamHI: CGGGATCCGCAGAGCTGGTTTAGTGAACCGTCAG	EcoRI: CGGAATTCTCACCAAAGATGAGTCTCCCGGATTTC
Lenti-Cry2-mCherry-Drp1	pFCK(1.3)GW	Cry2-mCherry-Drp1	BamHI: CGGGATCCGCAGAGCTGGTTTAGTGAACCGTCAG	EcoRI: CGGAATTCTCACCAAAGATGAGTCTCCCGGATTTC
pGAD-Mid49	pGAD10	pcDNA3.1 (-) MID49 4xMyc Hisx6	XhoI: GCCGCTCGAGGAATGAAGCGGCTCATCGACAGGGC	EcoRI: CGGCGGAATTCCTAGAATAGGCTTTCAGGAACCTG
pGAD-Mid51	pGAD10	pcDNA3.1 (-) MID51 4xMyc Hisx6	XhoI: GCCGCTCGAGGAATGAAGCGGATGTATGACCGGGC	EcoRI: CGGCGGAATTCCTACGTCTGCAGCAGCACCTCCG

pGAD-Mff	pGAD10	pcDNA3 Flag MFF iso4 WT	XhoI: GCCGCTCGAGGAATGGCAGAAATTAGTCGAATTC	EcoRI: CGCGGATCCCTATGACGTTCCTTCAATGGTTG
pBHA-Mid49	pBHA	pcDNA3.1 (-) MID49 4xMyc Hisx6	EcoRI: CGCCGGAATTCATGAAGCGGCTCATCGACAGGGC	BamHI: CGCGGATCCCCTAGAATAGGCTTTCAGGAACCTG
pBHA-Mid51	pBHA	pcDNA3.1 (-) MID51 4xMyc Hisx6	EcoRI: CGCCGGAATTCATGAAGCGGATGTATGACCGGGC	BamHI: CGCGGATCCCCTACGTCTGCAGCAGCACCTCCG
pBHA-Mff	pBHA	pcDNA3 Flag MFF iso4 WT	SmaI: TCCCCCGGGGATGGCAGAAATTAGTCGAATTC	SalI: CCGACGTCGACGCTATGACGTTCCTTCAATGGTTG
pBHA-dysbindin-1c	pBHA	GW1-Dys1C	EcoRI: CGCCGGAATTCATGCTGTCTGCCCACTGG	BamHI: CGCGGATCCCTTAAATGTCCTGAGTTGAG

Lentivirus production. HEK-293T cells (purchased from ATCC, not authenticated, not tested for mycoplasma contamination) were cultured on 15 cm plates coated with 0.1% gelatin in DMEM media supplemented with 10% FBS (Thermo Fisher Scientific). When the cell reached 90% confluence, the medium was changed at 2 hours before transfection. For transfection of each 15-cm plate, 22 μg pFCK(1.3)GW containing genes of interest, 15 μg psPAX2, 5 μg pMD2.G and 2 μg pAdVantage plasmids were added to 2 ml water containing 260 μl $CaCl_2$ (2 M), and then mixed with 2 ml 2X HBSS (50mM HEPES, 280 mM NaCl, 1.5 mM Na_2HPO_4, pH 7.05). After incubation at room temperature for 2 min, the mixture was added to the culture plate dropwise. The medium was replaced with 15 ml UltraCULTURE medium (UltraCULTURE, 1mM Sodium Pyruvate, 0.075% Sodium Bicarbonate, 1x Glutamine) at 16 hr after transfection. The medium was removed 48 hr after transfection and kept at 4°C. 15 ml fresh UltraCULTURE medium was added to the plate and collected 72 hr after transfection. The media collected at the two times were combined, filtered with 0.45 μm filter bottles, and centrifuged at 25,000 rpm for 90 min at 4°C (Beckman, SW28 rotor). The supernatant was removed and the pellet containing the virus was dissolved by incubation with 100 μl 1X HBSS overnight at 4°C. The viral suspension was placed on top of 1.5 ml 20% sucrose (in 1X HBSS) and centrifuged at 21,000 rpm for 2 hours at 4°C (Beckman, SW55 rotor). The pellet was incubated with 100 μl 1X HBSS overnight at 4°C, aliquoted and stored at -80°C. The titer of purified virus was determined by transducing HEK-293T cells with a series of dilutions. All viruses used for *in vivo* injection had a titer of 10^9–10^{10} IU/ml.

Neural culture, glia culture, and transfection. Neurons were seeded on coverslips coated with 30 μg/ml poly-D-lysine and 5 μg/ml laminin at a density of 330 cells/mm^2 and grown in Neurobasal media (Thermo Fisher Scientific) supplemented with 2% B27 (Thermo Fisher Scientific), 1% penicillin-streptomycin and 1% glutamax (Thermo Fisher Scientific). For glial cultures, dissociated hippocampal cells from mouse embryos (E18-19) were cultured in DMEM medium.

Organotypic hippocampal slice culture. Hippocampal slices were cultured from WT or sdy mice (6–8 days of age) as described [4]. 400-μm thick horizontal slices were cut with a vibratome (Leica VT1000S), placed on semipermeable membrane inserts (Millipore) sitting on a 6-well plate, and cultured with 1 ml medium (78.8% MEM, 20% heat-inactivated horse serum, 26 mM $NaHCO_3$, 25 mM HEPES, 10 mM D-glucose, 2 mM $CaCl_2$, 2 mM $MgSO_4$, 0.0012% ascorbic acid, 1 μg/ml insulin; pH 7.3; 320–330 mOsm). The medium was changed every 2 days.

Acute hippocampal slices. Mice were anesthetized by isoflurane overdose. The brain was removed and submerged in ice-cold cutting buffer (238 mM sucrose, 2.5 mM KCl, 26 mM $NaHCO_3$, 1 mM NaH_2PO_4, 5 mM $MgCl_2$, 11 mM D-glucose and 1 mM $CaCl_2$, bubbled with 95% O_2 / 5% CO_2, pH 7.4). 400-μm thick horizontal brain slices were cut in ice-chilled cutting buffer with a Leica VT1000S vibratome. Immediately after cutting, brain slices were incubated in NMDG-HEPES recovery solution (93 mM NMDG, 2.5 mM KCl, 1.2 mM NaH_2PO_4, 30 mM $NaHCO_3$, 20mM HEPES, 25 mM D-glucose, 5 mM sodium ascorbate, 2 mM thiourea, 3 mM sodium pyruvate, 10 mM $MgSO_4$, 0.5 mM $CaCl_2$, bubbled with 95% O_2 / 5% CO_2, adjust pH to 7.4 with HCl) for 15 min at 33°C, and then

transferred to normal ACSF at room temperature to incubate for at least 1 hr before whole-cell recordings or 2 hr before field potential recordings.

***In vitro* electrophysiological recording.** For the recording of field potentials, hippocampal slices were recovered in a chamber filled with ACSF for ≥ 2 hours at room temperature after cutting and then transferred to an interface recording chamber maintained at 28–32°C for 30 min before recording. Recording electrodes were glass pipettes filled with ACSF (1–2 MΩ) and placed at the stratum pyramidale of the CA3 region ~200 μm below the surface. Field potentials were amplified with an Axon Multiclamp 700B amplifier, digitized at 10 kHz with an Axon Digidata 1440A, and low-pass filtered at 2 kHz. Power spectra, time-frequency spectrogram, and integrated power at 20–80 Hz were analyzed using the Fast Fourier Transformation function of the Chronux toolbox in Matlab.

Whole-cell recordings were performed in a submerged chamber perfused with ACSF at a rate of 2 ml/min. Recording pipettes (5–7 MΩ) were filled with the intracellular solution (130 mM K-gluconate, 10 mM KCl, 2 mM $MgCl_2$, 10 mM HEPES, 2 mM Mg-ATP, 0.2 mM Tris-GTP, adjusted to pH 7.2 with KOH, 280 mOsm). The electrical signals were amplified with Axon Multiclamp 700B and digitized at 10 kHz with Axon Digidata 1440A. In hippocampal slices, current-clamp mode was used to record membrane potentials of visually identified CA3 pyramidal neurons. The firing probability of neurons in response to stimulations at various frequencies was analyzed by applying 1-min trains of current pulses (1 ms pulse duration) at 20, 30, 40, 50, or 80 Hz. Series resistance (Rs) was monitored during recording. Only neurons with Rs < 30 MΩ, action potential amplitudes >

70 mV, resting membrane potentials < -50 mV, and the drift of resting membrane potential during the recording period less than < 10% were included in the analysis. For recordings of primary hippocampal neurons, neurons were transfected with ChR2 at DIV14 and recorded at DIV17–19. After break-ins, only neurons with a resting membrane potential below -55 mV were used for further analysis. Optical stimulation-elicited EPSCs were recorded by illuminating cells held at -70 mv in voltage-clamp mode with 473 nm-light pulses (40 Hz, 1 ms pulse duration, 2 mW/mm^2). Light pulses generated from a laser (CrystaLaser) controlled by a Master-8 Pulse Stimulator (A.M.P.I.) were delivered to the cell through an optical fiber (200 μm in diameter).

Live imaging and light stimulation. For the assessment of mitochondrial dynamics, cultured hippocampal neurons or hippocampal slices were placed in a chamber mounted on the sample stage of an Olympus FV1000 confocal microscope, perfused with ACSF at a rate of 2 ml/min at 30°C. Images were acquired with a 60X objective (NA = 1.0) every 15 s. For ChR2 stimulation, an optical fiber (NA = 0.39, ThorLab FT200EMT) connected to a 473-nm laser (CrystaLaser controlled by a Master-8 Pulse Stimulator) was inserted into the imaging chamber. After baseline imaging for 10 min, light pulses (40 Hz, 2 mW/mm^2, 1 ms pulse duration) were applied while imaging acquisition continued. For light induced-CIB1 and Cry2 interaction in primary hippocampal neurons, continuous light (488 nm, ~1 mW/mm^2) was delivered through the objective for 10 min using the bleach function of the Fluoview software. For light-induced CIB1 and Cry2 interaction in electrophysiological experiments, continuous light stimulation (generated by a 473-nm laser, 2 mW/mm^2) was delivered through an optical fiber placed at a ~ 60° angle, ~500 μm above the recorded region.

Immunostaining. Primary hippocampal neurons were fixed in PBS containing 4% formaldehyde and 4% sucrose for 12 min at room temperature. After the rinse, neurons were incubated with primary antibodies diluted in GDB buffer (0.1% gelatin, 0.3% Triton X-100, 16 mM sodium phosphate, 450 mM NaCl, pH 7.4,) at 4 °C overnight, washed with PBS for three times, incubated with Alexa Fluor dye-conjugated secondary antibodies at room temperature for 1 hour, washed with PBS for three times, and then mounted with mounting media (Vectorlabs, H1000).

Image acquisition and image analysis. An Olympus Fluoview 1000 confocal microscope (with a 60X, NA 1.0 objective) was used for live imaging and a Zeiss 510 confocal microscope (with a 63X, NA 1.4 objective) was used for fixed samples. All z-stack confocal images were converted to Tiff files using the Bio-Formats plugin of Image J. Salt-and-pepper noise was removed using a median filter with radius 1 in Image J. For the analysis of mitochondrial morphology, 2D projections of z-stack images were used to identify individual mitochondria using the Otsu thresholding method in Image J. The area and length of mitochondria in secondary dendrites were measured using the Analyze Particles function in ImageJ (Feret's diameter was used for mitochondrial length). To analyze mitochondrial area, the total area of all mitochondria in a dendritic segment was divided by the total length of the dendrite to obtain the mean mitochondrial area (μm^2) per μm dendrite. When analyzing mitochondrial length in slices, because the density of transduced dendrites was high, it was difficult to assign individual dendrites to specific neurons. We, therefore, measured the length of mitochondria in clearly identified dendrites to obtain the mean mitochondrial length for each slice (~30-50 mitochondria per slice). When analyzing mitochondrial length in primary hippocampal neurons, because of

the medium cell density and sparse transfection, we were able to trace the dendrites of transfected neurons. All mitochondria in the secondary dendrite of a transfected neuron in the imaged field (~30-50 mitochondria) were measured to obtain the average mitochondrial length of this neuron.

For mitochondrial fission and fusion, 2D projections of time-lapse images were analyzed by visual inspection. Fission events were defined as the split of one mitochondrion into two, while fusion was coalescing of two mitochondria into one that stayed as one structure for at least 2 min. The fission and fusion rates were calculated as the number of events observed in a 10 μm dendritic segment per min.

For analysis of endogenous Drp1 puncta, z-stack images collected at 0.1-μm thickness using a Zeiss 510 microscope with a 63X objective (NA 1.4) were deconvoluted with MetaMorph software using the 3D Deconvolution function and the blind algorithm to estimate the point spread function. Individual Drp1 puncta were separated with the Otsu thresholding method and measured with the Analyze Particles function in Image J. The size of Drp1 puncta was calculated as the area of Drp1 puncta, and the density of Drp1 puncta was calculated as the number of Drp1 puncta on mitochondria.

For analysis of dendritic spines, z-stack confocal images were imported into Neurolucida 360 software (MBF Bioscience). Dendritic spines from 1–3 secondary dendrites of a transfected neuron within the imaging field were measured and classified with Neurolucida 360 as reported [5-7].

For analysis of mitochondrial motility, a mitochondrion that moved more than its length in the first image during the 30 min imaging period was identified as a motile mitochondrion.

The percentage of motile mitochondria in the secondary dendrites or axons was calculated by dividing the number of motile mitochondria by the total number of mitochondria. The speed of a mitochondrion was calculated by dividing its net displacement during the 30 min imaging period by time.

For analysis of TMRE and MitoSOX fluorescence. Z-stack confocal images were collapsed using the “Max intensity z projection” function in ImageJ. For brain slices, fluorescence intensity in the CA3 stratum pyramidale was measured. For cultured neurons, a transfected neuron was outlined using the plugin “Find Edges” in ImageJ, and fluorescence intensity was measured. The pseudo color was generated with the "Lookup tables” function in ImageJ.

Preparation of the mitochondrial fraction. The hippocampus (~35 mg) or cultured hippocampal neurons (~300,000) were homogenized in 400 μl fresh ice-cold MB buffer (210 mM mannitol, 70 mM sucrose, 1 mM EDTA, 10 mM HEPES, 1 μg/ml aprotinin, 1 μg/ml leupeptin, 1 mM PMSF, pH 7.4) on ice using 20 strokes with a Pellet Pestle Motor (Kontes), followed by centrifugation at 2,000 g for 10 min at 4°C. The supernatant was centrifuged again at 13,000 g for 30 min at 4°C. The pellet was washed by resuspension in MB buffer (1 ml) and centrifugation at 13,000 g for 30 min at 4°C. After wash, the pellet was resuspended in 200 μl ice-cold 3% Ficoll solution, layered onto 800 μl ice-cold 6% Ficoll solution, and then centrifuged at 11,500 g for 25 min at 4°C. The mitochondrial fraction (pellet) was washed in 1 ml MB buffer, centrifuged at 12,000 g for 10 min at 4 °C, and then dissolved in 100 μl RIPA buffer (50 mM Tris-HCl pH8.0, 150mM NaCl, 0.1%

Triton X-100, 0.1% SDS, 0.5% sodium deoxycholate, 1 μg/ml aprotinin, 1 μg/ml leupeptin, 1 mM PMSF).

Preparation of the PSD fraction. Brain tissue was homogenized manually using a Dounce homogenizer with 12 strokes in 2 ml Solution A (0.32 M sucrose, 1 mM $NaHCO_3$, 1mM $MgCl_2$, 0.5 mM $CaCl_2$) on ice. The homogenate was diluted with Solution A to obtain a 10% weight/volume percentage, followed by centrifugation at 710 g for 10 min. The pellet was resuspended in Solution A by homogenization in a Dounce homogenizer with 3 strokes, spun at 1400 g for 10 min. The supernatants from the two centrifugations were pooled (S1) and centrifuged at 13,800 g for 10 min. The supernatant (S2) was saved for immunoblotting. The pellet (P2) was homogenized with 6 strokes in Solution B (0.32 M sucrose, 1 mM $NaHCO_{3;}$ 1.6 ml per 0.5 g tissue). 2 ml of homogenized P2 was placed on top of a discontinuous sucrose gradient consisting of 1.2 M, 1.0 M, 0.85 M sucrose (2.5 ml for each concentration), and centrifuged at 82,500 g, 4 ^{o}C for 2 hr. Synaptosomes, enriched between 1.0 M and 1.2 M sucrose, were removed with a glass pipette. The synaptosome fraction was resuspended in Solution B (3 ml/0.5 g tissue) and then incubated with an equal volume of solution C (1% Triton X-100, 0.32 M sucrose,12 mM Tris-HCl, pH 8.1) on ice for 15 min, followed by centrifugation at 35,000g for 20 min. The pellet was incubated with 3 ml Solution C for 15 min on ice, and then centrifuged at 201,800 g for 1 hr to obtain the PSD fraction.

Cross-linking. The hippocampus or hippocampal neurons were lysed in MB buffer and then cross-linked with DSP [dithiobis (succinimidyl propionate), at 1 mM final concentration] on ice. The reaction was quenched by adding Tris-HCl (pH 7.5, final

concentration 100 mM) and incubation on ice for 15 min. The crosslinked lysate was diluted with SDS-sample buffer without reducing agents for immunoblotting.

Immunoprecipitation. 10 μl Protein G Sepharose Fast Flow 4 beads (GE # 17600235) slurry was pre-incubated with 2 μg antibodies for 2 hours at 4°C, followed by washing with TBST (50 mM Tris, 150 mM NaCl, 0.1% Tween 20, pH 7.4) four times. The antibody-coated bead was incubated with 0.1–1 mg cell lysates overnight at 4°C, washed three times with TBST, eluted with SDS-sample buffer, boiled, and separated by gel electrophoresis with 4–12% Bis-Tris protein gels (Invitrogen, NuPAGE Novex) or 4–15% mini-protean precast gels (Bio-Rad) for immunoblotting.

GTPase activity of Drp1. GTPase activity of Drp1 was measured by using a GTPase Assay Kit (Novus Biologicals) according to the manufacturer's instructions. Briefly, Drp1 was purified from 1 mg whole-cell lysate by immunoprecipitation with the anti-Drp1 antibody followed by washing with lysis buffer for three times and the GTPase buffer (50 mM Tris, 2.5 mM $MgCl_2$, 0.02% 2-mercaptoethanol, pH 7.5) for three times. Purified Drp1 was diluted in 100 μl water and incubated with 100 μl substrate mix at room temperature for 30 min in a 96-well plate. The enzymatic reaction was stopped by adding 50 μl phosphate detection reagent PiColorLock™. After 2 min incubation in the PiColorLock™ mix, 20 μl Stabilizer was added, and the plate was read at 650 nm with Perkin Elmer Victor3.

Yeast two-hybrid. The bait and prey plasmids were transformed into L40a yeast that contains LexA operators upstream of the HIS3 and lacZ reporter genes using a Yeast Transformation kit (Takara). The transformation mixture was plated on Trp /Leu drop out

SD plates to select for diploid cells containing both the Trp1-carrying pBHA and Leu2-bearing pGAD10 plasmids. The plate was incubated at 30°C for 2–3 days. The diploids were picked up, diluted in water, measured for OD600, and made into a series of 10-fold dilutions. The diluted yeast was plated on His/Leu/Trp drop out SD plates, and grown at 30°C for 2–3 days.

Electron microscopy. After light illumination, neurons grown on glass coverslips were immediately fixed with 4% paraformaldehyde and 2% glutaraldehyde in 0.1 M phosphate buffer (pH 7.4, pre-warmed to 37°C) for 15 min at 37°C, and then at 4°C for 30 min. Neurons were washed for 3 times with PBS at 4°C (10 min each), followed by washing with 0.1 M cacodylate buffer (pH 7.4) for 3 times (5 min each), and then incubated with 1% osmium tetroxide in 0.1 M cacodylate buffer for 30 min at room temperature (in the dark). After rinsing with cacodylate buffer for 3 times (5 min each), neurons were dehydrated in ethanol with the following order: 3×5 min in 50% ethanol, 1×10 min in 50% ethanol containing 1% uranyl acetate, 2×5 min in 75% ethanol, 1×10 min in 95% ethanol, 3×10 min in 100% ethanol. Residual ethanol was removed by rinsing with propylene oxide (PO) twice. The coverslip was embedded in Epon:PO (1:1) for 1 hr at room temperature, and then embedded in pure Epon overnight. On the second day, the coverslip was transferred to fresh Epon for 1 day, and then baked in the oven for 2 days at 64°C. The glass coverslip was removed by incubation with hydrofluoric acid for 30 min. The Epon block containing neurons was trimmed and then cut into thin sections (80 nm) using a Leica UC7 Ultramicrotome (Vienna), and the thin sections were then stained with lead citrate. A JEOL JEM-2100 transmission electron microscope (Peabody, MA) coupled with a Gatan digital camera (Pleasanton, CA) was used to take micrographs of randomly

selected regions by an experimenter blind to the treatment and genotype of the sample. 90–120 micrographs were taken from each coverslip. The postsynaptic density was identified as an electron-dense region at the membrane of a postsynaptic neuron and in close apposition to the presynaptic neurotransmitter release sites which contain synaptic vesicles. These discernible synapses in the electron micrographs were identified for the analysis of mitochondria, and mitochondria within 500 nm of the PSD were classified as mitochondria near PSD.

NOR test. The NOR test had two sessions, a sample session and a test session. The two sessions were separated by 6 hr. During the sample session, the mice were placed in a test box containing two identical objects and allowed to freely explore the box for 10 min. The mouse was returned to its home cage after the sample session and placed in the test box again 6 hr later for the test session. During the test session, one object remained the same, the other object was replaced by a new one, and the mouse was allowed to explore the test box for 10 min. At one week after the first NOR test, the mouse was subjected to the second NOR test. Only objects that the mouse had not seen in the first NOR test were used in the second NOR test. Mouse behavior was recorded with a video camera at a rate of 50 frames per second. Behavioral data were analyzed manually. To avoid the experimenter bias, all video files were relabeled by individuals who were blind to the treatment, and the researcher analyzing the video remained blind to the animal's identity throughout the analysis. Exploration was defined as directing the nose towards the object within a 2-cm distance for at least 300 ms without climbing over the object. Explorations during the first period that the total exploration duration reached 15 seconds were used to calculate the ratio of exploration time spent on the novel and familiar objects. Mice that

did not reach the 15-second criterion during the 10-min test session were excluded from the analysis.

Surgery. Mice were anesthetized with isoflurane (5% for induction and 1.5% for maintenance) and placed on a stereotaxic frame. A small craniotomy was made and 1 μl lentivirus was injected into the CA3 region using a 5 μl gas-tight Hamilton Syringe (32-gauge, flat-tip needle) mounted on a stereotaxic device at a rate of 0.2 μl/min. For electrophysiological recording in hippocampal slices, the following coordinates were used: AP, -2 mm; ML, ±1.9 mm; DV, -1.8–1.5 mm. The mouse was returned to the home cage for recovery after viral injection. At 3–4 hours after viral injection, two optic fibers (one on each side of the brain; 200 μm in diameter; for the NOR test) or a microdrive-controlled optoelectrode (for *in vivo* recording) which contained 8 tetrodes (with an impedance of 150-400 kΩ measured at 1000 Hz) and an optical fiber (200 μm in diameter) was implanted 100 μm above the viral injection site. The craniotomy was sealed with bone wax. Two stainless steel screws connected with a copper wire were inserted into the contralateral skull to be used as the ground and reference for recordings. Several additional screws were inserted into the skull and glued to the microdrive using dental cement. A copper mesh was attached to the skull with dental cement and connected to the ground screw to act as a Faraday cage to block the external electrical noise.

***In vivo* recording.** *In vivo* recording began at one week after surgery by using an Intan 32-channel RHD2000 evaluation system. During the first week of recording, the optoelectrode was lowered through the microdrive with a daily increment of ~70 μm until the electrode tip reached the stratum pyramidale of the CA3 region as indicated by the

appearance of spikes from putative excitatory neurons. The mice were then subjected to two NOR tests and recorded during the test session at a sampling rate of 30 kHz. During all recording sessions, the mouse behavior was recorded with a camera synchronized with electrophysiological recordings. At 5 hr after the sample session of the second NOR test, the mouse was anesthetized with 5% isoflurane and stimulated through the implanted optic fiber (473 nm, 10 min, 2 mW/mm^2). The mouse recovered from anesthesia after photostimulation and were subjected to the test session of the second NOR test one hour later.

For the analysis of *in vivo* recording data, LFPs were separated by applying the 1–250 Hz butterworth band-pass filter and the 60-Hz notch filter. The electrode with the largest power of LFP oscillations during object exploration from each animal was selected for statistical analysis across animals. To reduce the effect of variations in individual animals' locomotor activities on the measurement of LFP oscillations during object exploration, we calculated the ratio of LFP oscillations before and during object exploration. To this end, LFP epochs during object exploration and those recorded during the same duration of time after object exploration were extracted for the spectral analysis with the Multitaper method using the Chronux toolbox in Matlab. The time-frequency spectrogram was generated using the *mtspecgramc* function in the Chronux toolbox with the following parameters: fpass = [0,100], movingwin = [0.5,0.05], tapers = [3,5]. Based on the inflection point of humps on the power spectral density (PSD) curve, gamma-band was defined as 30–80 Hz and theta band as 6–15 Hz for analysis. Integrated PSD in these ranges was calculated to indicate the power of LFP oscillations. The ratio of integrated PSD after and before object exploration was used for comparison across animals.

Statistical analysis. Based on our previous studies, we determine that the following sample sizes are appropriate: 5-12 cells for live imaging, 8-15 cells for immunofluorescence in cultured neurons, 3-6 biological replicates for immunoblotting; 6-12 slices for electrophysiology; 3-5 animals for in vivo electrophysiology; 8-15 animals for behavioral testing. For comparison of two groups, data were analyzed for normality and variance and then subjected to two-tailed Student's t-test and two-tailed paired Student's t-test for normally distributed data with equal variance, or two-tailed Mann-Whitney U test and two-tailed Wilcoxon-signed rank test if the normality and equal variance tests failed. For comparison of ≥ 3 groups, one or two-way ANOVA (for data that satisfy the normality assumption) or one or two-way ANOVA on ranks (for data that do not) was used, and Holm-Sidak (for multiple comparisons), two-tailed Student's t-test or two-tailed Mann-Whitney U test was used for comparison between two groups. $P < 0.05$ was considered significant. Animal assignment to individual experimental conditions was random. The experimenters were blind to experimental conditions during data collection and data analysis. All animal assignment and data analyses were done blindly to the experimental condition.

References

1. Jia JM, Zhao J, Hu Z, Lindberg D, Li Z. Age-dependent regulation of synaptic connections by dopamine D2 receptors. *Nature neuroscience* 2013; **16**(11)**:** 1627-1636.

2. Jia JM, Hu Z, Nordman J, Li Z. The schizophrenia susceptibility gene dysbindin regulates dendritic spine dynamics. *J Neurosci* 2014; **34**(41)**:** 13725-13736.

3. Li Z, Okamoto K, Hayashi Y, Sheng M. The importance of dendritic mitochondria in the morphogenesis and plasticity of spines and synapses. *Cell* 2004; **119**(6)**:** 873-887.

4. Hu Z, Zhao J, Hu T, Luo Y, Zhu J, Li Z. miR-501-3p mediates the activity-dependent regulation of the expression of AMPA receptor subunit GluA1. *J Cell Biol* 2015; **208**(7)**:** 949-959.

5. Harris KM, Jensen FE, Tsao B. 3-Dimensional Structure of Dendritic Spines and Synapses in Rat Hippocampus (Ca1) at Postnatal Day-15 and Adult Ages - Implications for the Maturation of Synaptic Physiology and Long-Term Potentiation. *Journal of Neuroscience* 1992; **12**(7)**:** 2685-2705.

6. Peters A, Kaiserman-Abramof IR. The small pyramidal neuron of the rat cerebral cortex. The perikaryon, dendrites and spines. *Am J Anat* 1970; **127**(4)**:** 321-355.

7. Dickstein DL, Dickstein DR, Janssen WG, Hof PR, Glaser JR, Rodriguez A *et al.* Automatic Dendritic Spine Quantification from Confocal Data with Neurolucida 360. *Curr Protoc Neurosci* 2016; **77:** 1 27 21-21 27 21.

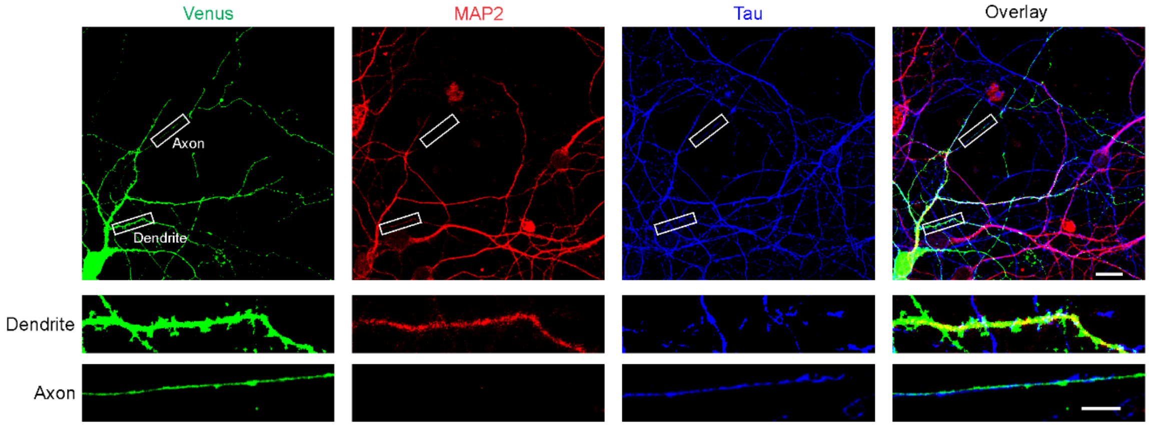

Supplemental Fig. 1. Dendritic and axonal markers in hippocampal neurons. Cultured hippocampal neurons (DIV17) were fixed 3 days after transfection with the Venus plasmid and stained with the anti-MAP2 (red) and anti-tau (blue) antibodies.

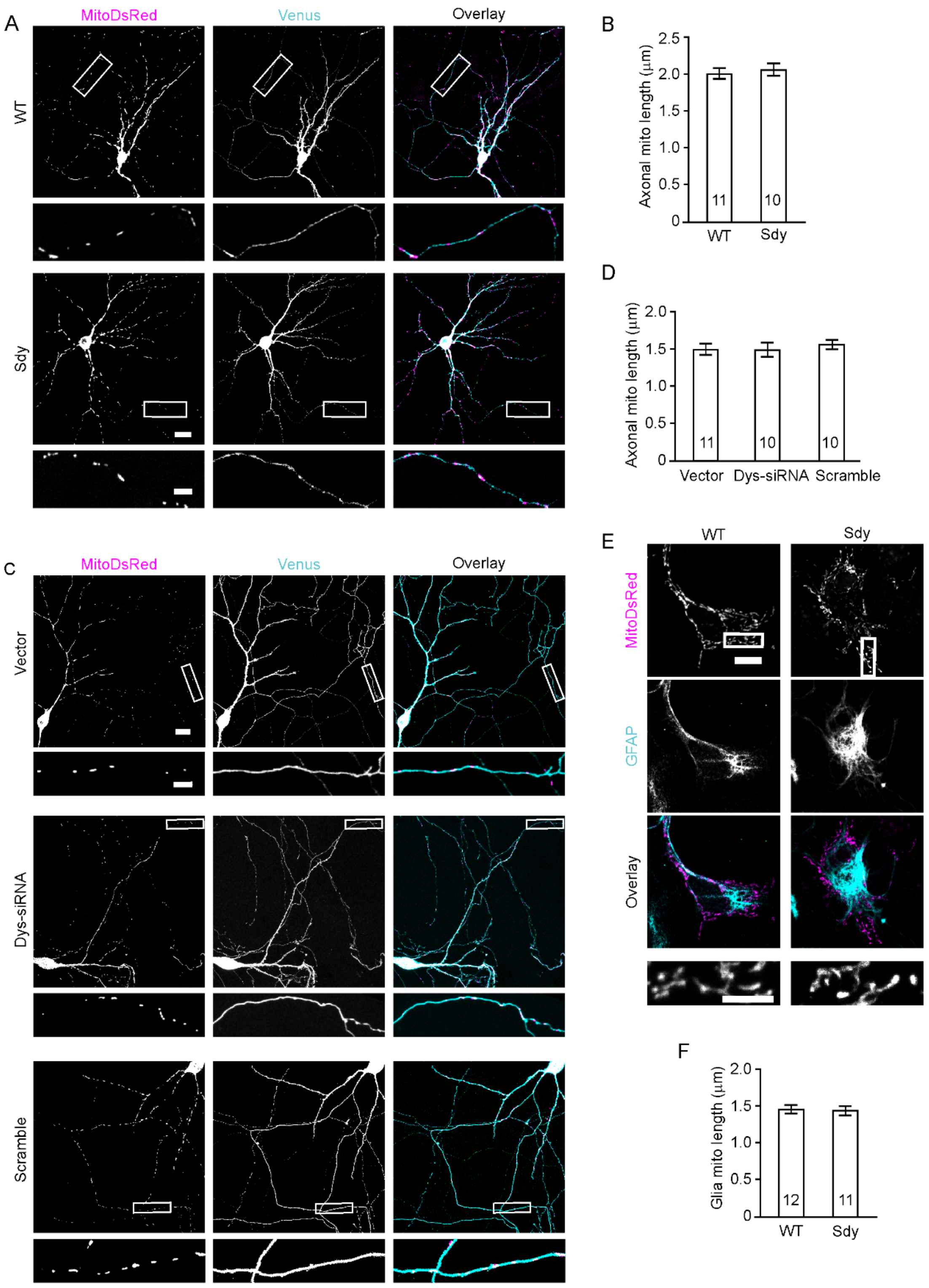

Supplemental Fig. 2. Mitochondrial length is unchanged in axons of neurons or glia with dysbindin-1 deficiency. Primary hippocampal neurons were transfected with the

mitoDsRed and Venus plasmids (A, B) or along with designated plasmids (C, D). (A, C) Representative images; scale bar, 20 μm for low-magnification and 5 μm for high-magnification. (B) Quantification for A; Student's t-test was used for statistical analysis. (D) Quantification for C; one-way ANOVA was used for statistical analysis, $F(2, 28) = 0.095$, $p = 0.91$. (E, F) Glial cells were fixed 3 days after transfection with the mitoDsRed plasmid and stained with the GFAP antibody. (E) Representative images; scale bar, 10 μm for low-magnification images and 5 μm for high-magnification images. (F) Quantification for E; Student's t-test was used for statistical analysis. Data are presented as mean ± SEM; n in the bar indicates the number of cells.

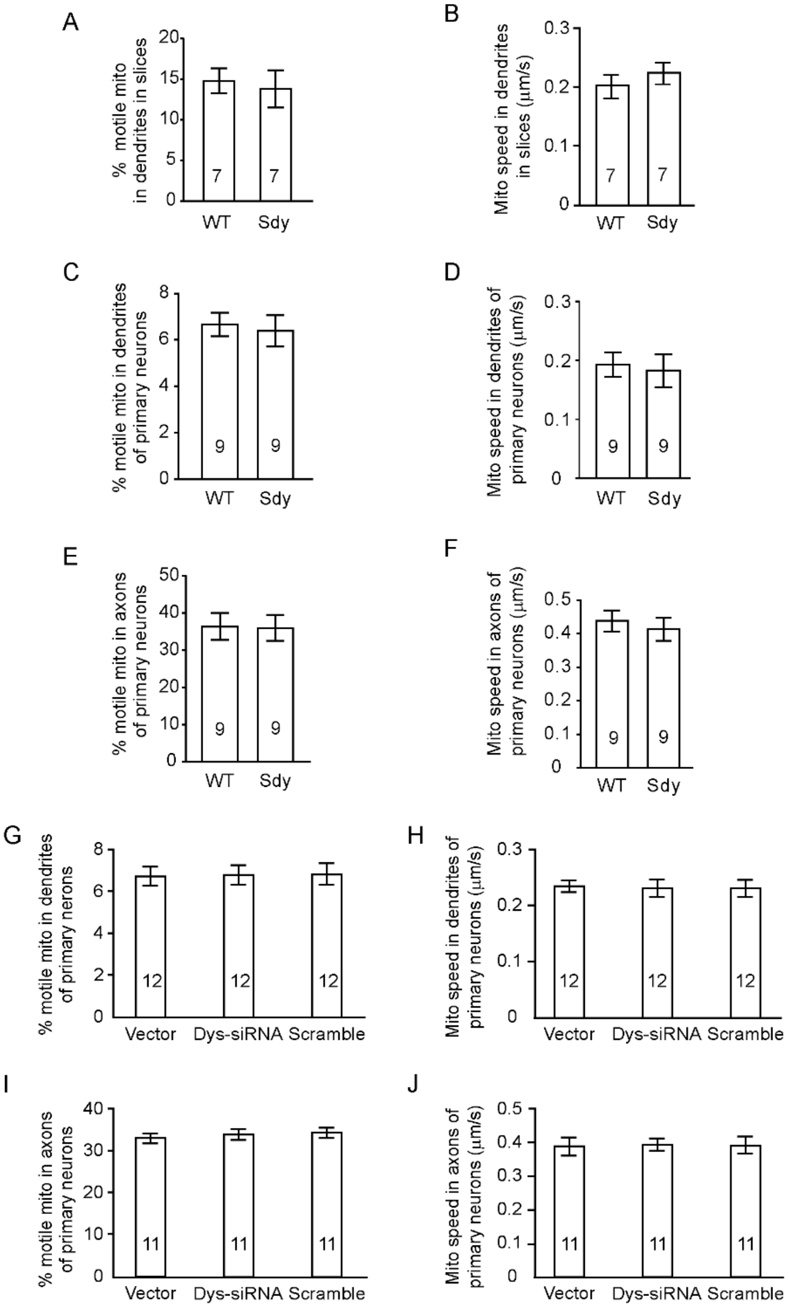

Supplemental Fig. 3. Dysbindin-1 deficiency has no effect on mitochondrial motility. Primary hippocampal neurons were transfected with the mitoDsRed and YFP plasmids and used for time-lapse imaging of mitochondria. Mitochondria with a net displacement of ≥ their length during the imaging period were designated as motile mitochondria. Mitochondrial speed was calculated by dividing the net displacement of the mitochondrial

centroid by mitochondrial travel time. (A, B) Quantification of dendritic mitochondrial motility in hippocampal slices. (C, D, G, H) Quantification of dendritic mitochondrial motility in primary hippocampal neurons. (E, F, I, J) Quantification of axonal mitochondrial motility in primary hippocampal neurons. Student's t-test was used for statistical analysis in A–F. One-way ANOVA was used for statistical analysis in G–J; G: $F_{(2, 33)} = 0.001273$, $p = 0.9987$; H: $F_{(2, 33)} = 0.01576$, $p = 0.9844$; I: $F_{(2, 30)} = 0.1256$, $p = 0.8825$; J: $F_{(2, 30)} = 0.007946$, $p = 0.9921$. Data are presented as mean ± SEM; n in the bar indicates the number of slices from 4 mice in A, B, and the number of cells in C–J.

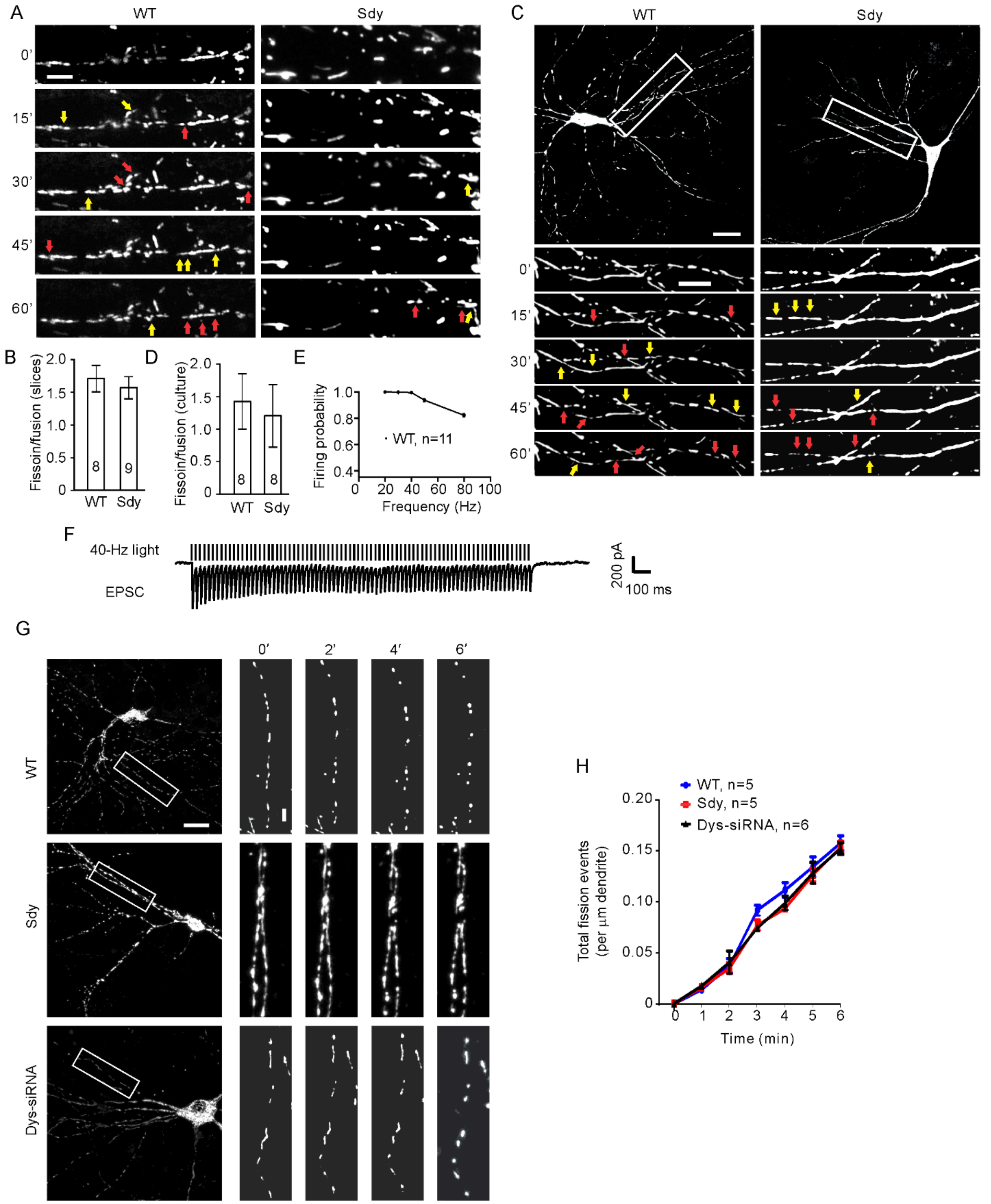

Supplemental Fig. 4. Mitochondrial fission and fusion balance after neurons have been transferred to the imaging chamber for 1 hr, the firing of hippocampal neurons

in response to electrical and optogenetic stimulations, and CCCP-induced mitochondrial fission. Cultured hippocampal slices from sdy mice and their WT littermates were transduced with lentivirus expressing mitoDsRed (A, B), or cultured hippocampal neurons transfected with the mitoDsRed plasmid alone (C, D) or along with the dysbindin-1 siRNA plasmid (G, H) were used for time-lapse imaging of mitochondria. (A) Representative time-lapse images of mitochondria in the CA3 region; mitochondrial fission sites were indicated by red arrows and mitochondrial fusion sites with yellow arrows; scale bar, 5 μm. (B) Quantification for A; Mann-Whitney U test was used for statistical analysis; n in the bar indicates the number of brain slices from 4 animals. (C) Representative time-lapse images of mitochondria in primary WT and sdy hippocampal neurons; mitochondrial fission sites were indicated by red arrows and mitochondrial fusion sites with yellow arrows; scale bar, 20 μm for upper and 10 μm for lower, enlarged images. (D) Quantification for C; Mann-Whitney U test was used for statistical analysis; n in the bar indicates the number of neurons. (E) Acute hippocampal slices were prepared from WT mice (7 weeks of age) for whole-cell recordings of CA3 pyramidal neurons in the current mode. Current pulses at 20, 30, 40, 50 and 80 Hz (100 pA, 10 s duration) were delivered to neurons. The histogram shows the average firing probability (the number of evoked action potentials divided by the number of stimulations) of 11 neurons. (F) Primary hippocampal neurons transfected with the ChR2 plasmid were recorded in the whole-cell mode for EPSCs elicited by light pulses (473 nm, 40 Hz, 1 ms pulse duration, 2 mW/mm^2). (G) Representative images of primary hippocampal neurons transfected with the mitoDsRed plasmid alone or along with the dysbindin-1 siRNA plasmid; CCCP (5 μM) was added to the perfusion solution after the first image (0 min) and present throughout

the imaging period; scale bar, 20 μm for upper and 5 μm for lower, enlarged images. (H) Quantification of the number of total fission events identified within 10 μm dendrite after CCCP treatment; two-way RM ANOVA was used to test for the influence of dysbindin deficiency on fission, $F_{(12, 78)} = 1.142$, $p = 0.34$; Data are presented as mean ± SEM.

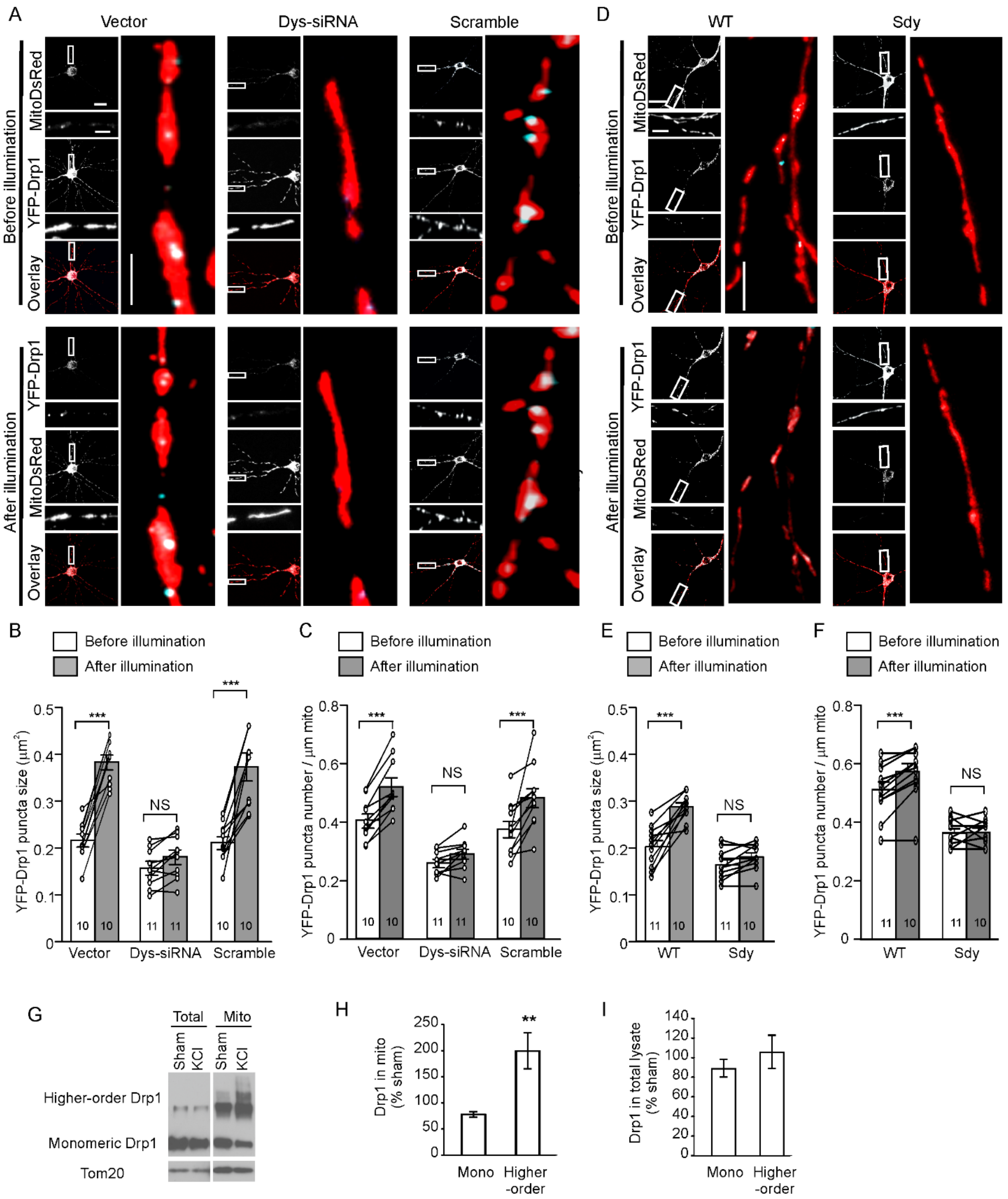

Supplemental Fig. 5. Dysbindin-1 is required for activity-induced Drp1 accumulation on mitochondria. Cultured hippocampal neurons were transfected with

designated plasmids and imaged before and after photostimulation in (A–F). (A, D) Representative images; scale bar: 20 μm for low-magnification images, 5 μm for high-magnification images. (B) Quantification for YFP-Drp1 puncta size on mitochondria; paired Student's t-test was used to compare the same cells before and after stimulation. (C) Quantification for YFP-Drp1 puncta number on mitochondria; paired Student's t-test was used to compare same cells before and after stimulation for the vector and the Dys-siRNA group; Wilcoxon signed rank test was used to compare same cells before and after stimulation for the scrambled group. (E, F) Quantification for YFP-Drp1 puncta size on mitochondria; paired Student's t-test was used to compare same cells before and after stimulation. (G–I) Cultured neurons were treated with 50 mM KCl in Tyrode's solution or sham-treated and harvested at 30 min after treatment for subcellular fractionation. (G) Representative blots. (H, I) Quantification for A; Student's t-test was used for B and Mann-Whitney U test was used for C; n = 5 experiments for each condition. Data are presented as mean ± SEM; n in the bar indicates the number of neurons; ** $p < 0.01$*** $p < 0.001$.

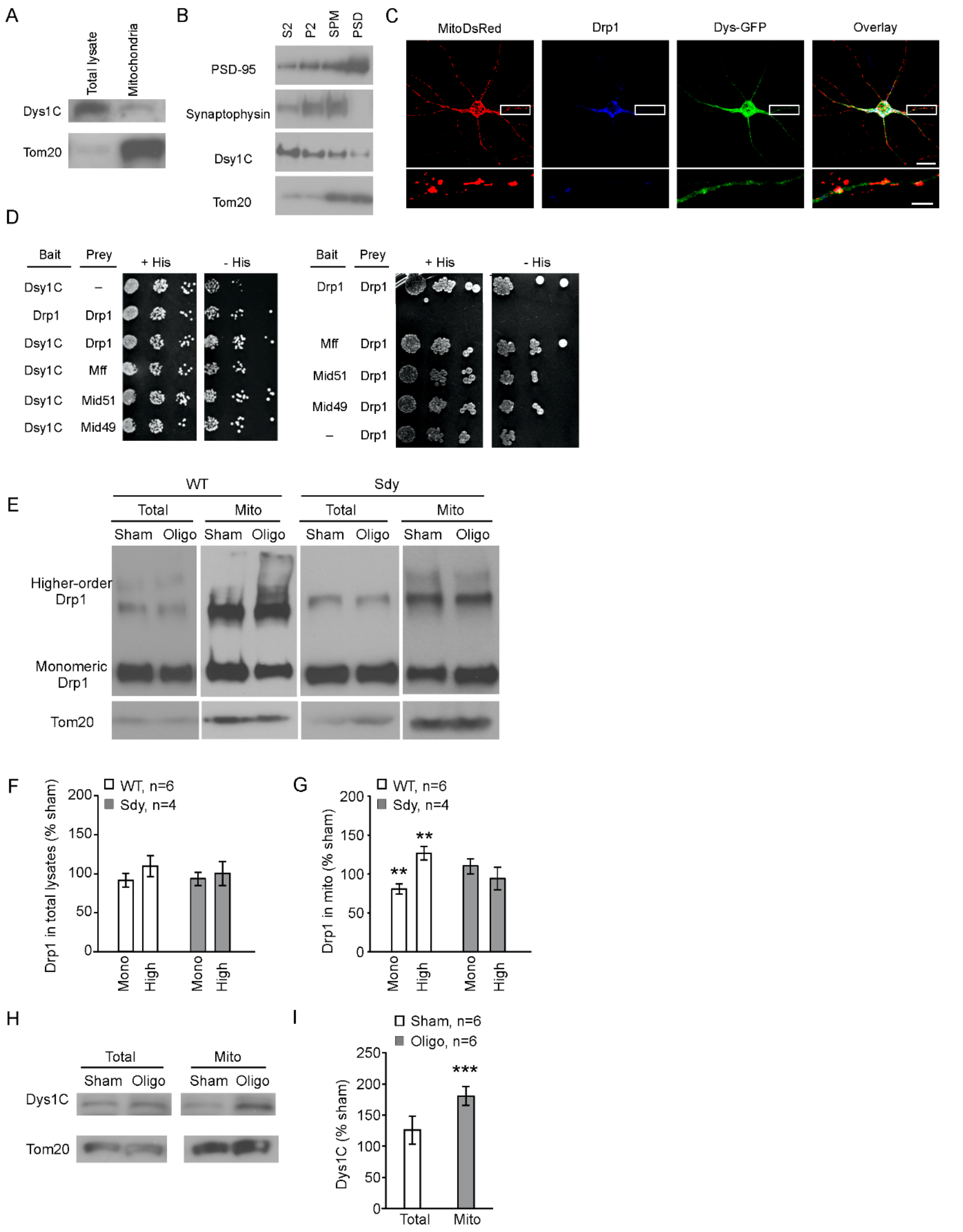

Supplemental Fig. 6. Mitochondrial and postsynaptic localization of dysbindin-1C, and the effect of oligomycin on dysbindin-1C and Drp1. (A, B) The mitochondrial and

PSD fraction were prepared from the hippocampus of WT mice (7-8 weeks of age). S2, the supernatant after the second centrifugation; P2, the pellet after the second centrifugation; SPM, synaptosome; PSD, postsynaptic density. (C) Primary hippocampal neurons were transfected with the mitoDsRed and dysbindin-1C-GFP plasmids and stained for Drp1 three days later; scale bar: 20 μm for low-magnification images, 5 μm for high-magnification images. (D) Yeast two-hybrid assay using the HIS3 reporter with the indicated bait and prey constructs are shown as sequential 10-fold yeast dilutions. (E–I) Hippocampal slices from WT and sdy mice (7-8 weeks of age) were sham-treated or treated with oligomycin (10 μM, 30 min), and then used for the preparation of total lysates and mitochondrial isolation. (E, H) Represent blots. (F, G) Quantification of monomeric Drp1 and higher-order Drp1 oligomers for D; One way ANOVA on ranks was used to compare across groups for Drp1 in the total lysate ($H = 1.957$, $DF = 5$, $p = 0.855$) and mitochondrial fraction ($H = 18.244$, $DF = 5$, $p = 0.003$); Mann-Whitney U test was used to compare Drp1 monomer and higher-order structures between sham and oligomycin treated samples. (I) Quantification for H; Mann-Whitney U test was used to compare Dys1C between sham and oligomycin treated samples. Data are presented as mean ± SEM; ** $p < 0.01$, *** $p < 0.001$; n indicates the number of experiments.

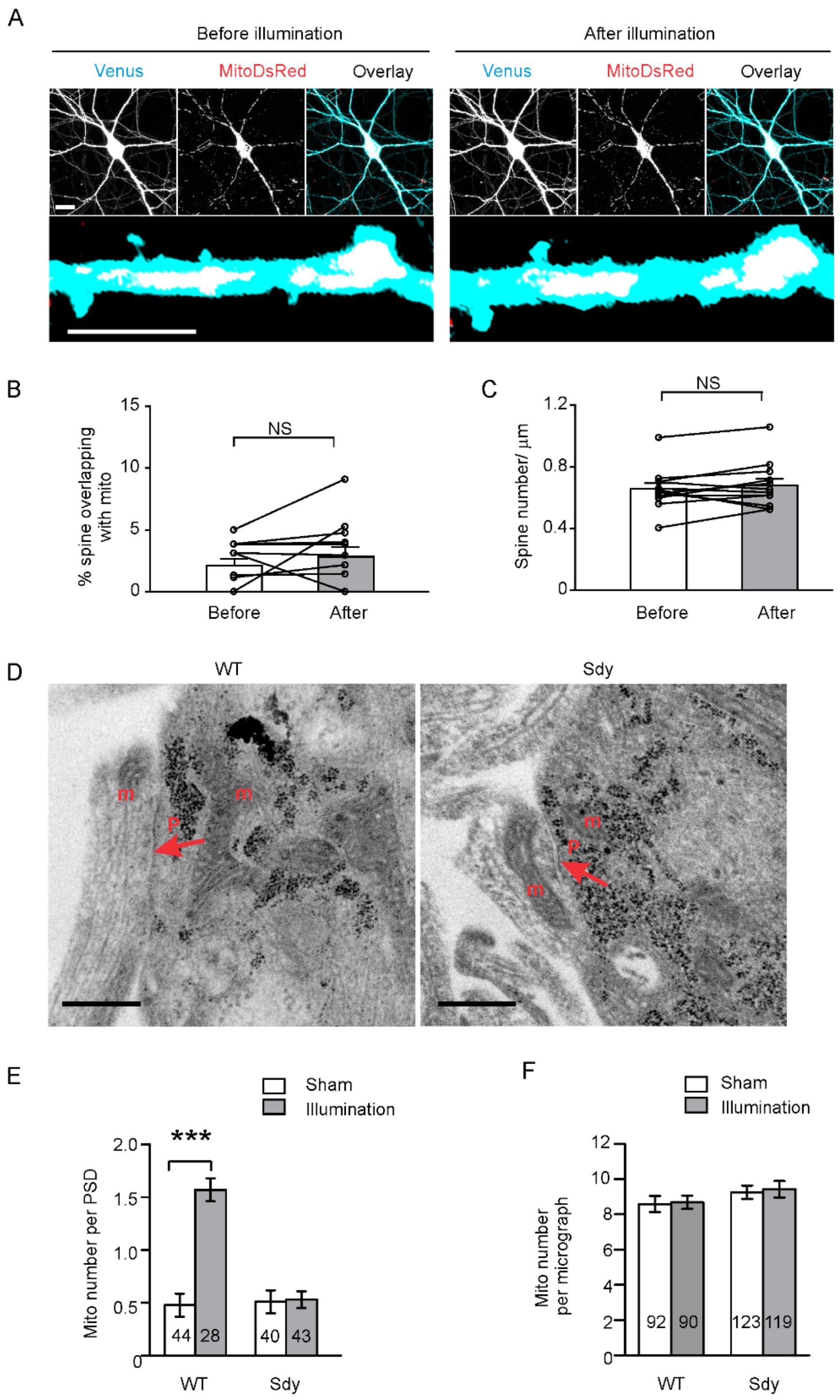

Supplemental Fig. 7. The effect of gamma-range stimulation on the localization of mitochondria in dendritic spines and postsynaptic sites. (A–C) Cultured hippocampal neurons were transfected with the ChR2, Venus, and mitoDsRed plasmids

and imaged before and after light stimulation (473 nm, 40 Hz, 10 min). (A) Representative images; scale bar, 20 μm in low-magnification and 5 μm in high-magnification images. (B, C) Quantification of A; Wilcoxon signed rank test was used to compare same cells before and after light stimulation for colocalization; paired Student's t-test was used to compare same cells before and after light stimulation for spine density. Data are presented as mean ± SEM; n =12 neurons. (D–F) Cultured WT and sdy hippocampal neurons were transduced with lentivirus expressing ChR2 and stimulated with light pulses (473 nm, 40 Hz, 10 min). (D) Representative electronic micrographs; m, mitochondria; p, PSD; arrows indicate PSDs; scale bar, 500 nm. (E) Mitochondria number within 500 nm of the PSD; n in the bar indicates the number of PSD; two-way ANOVA was used to test for the influence of genotype and light treatment on the number of mitochondria near the PSD. All effects were statistically significant ($p < 0.001$): the main effect for genotype, $F_{(1, 154)} = 25.386$; the main effect for light stimulation $F_{(1, 154)} = 31.648$; the interaction effect, $F_{(1, 154)} = 30.524$. Holm-sidak was used for post hoc multiple comparisons. (F) Mitochondria number in each electron micrograph; n in the bar indicates the number of micrographs; two-way ANOVA was used to test for the influence of genotype and light treatment on the number of mitochondria near PSD. All effects were not statistically significant: the main effect for genotype, $F_{(1, 423)} = 2.663$, $p = 0.103$; the main effect for light stimulation $F_{(1, 423)} = 0.0964$, $p = 0.756$; the interaction effect, $F_{(1, 423)} = 0.00595$, $p = 0.939$. Data are presented as mean ± SEM; *** $p < 0.001$.

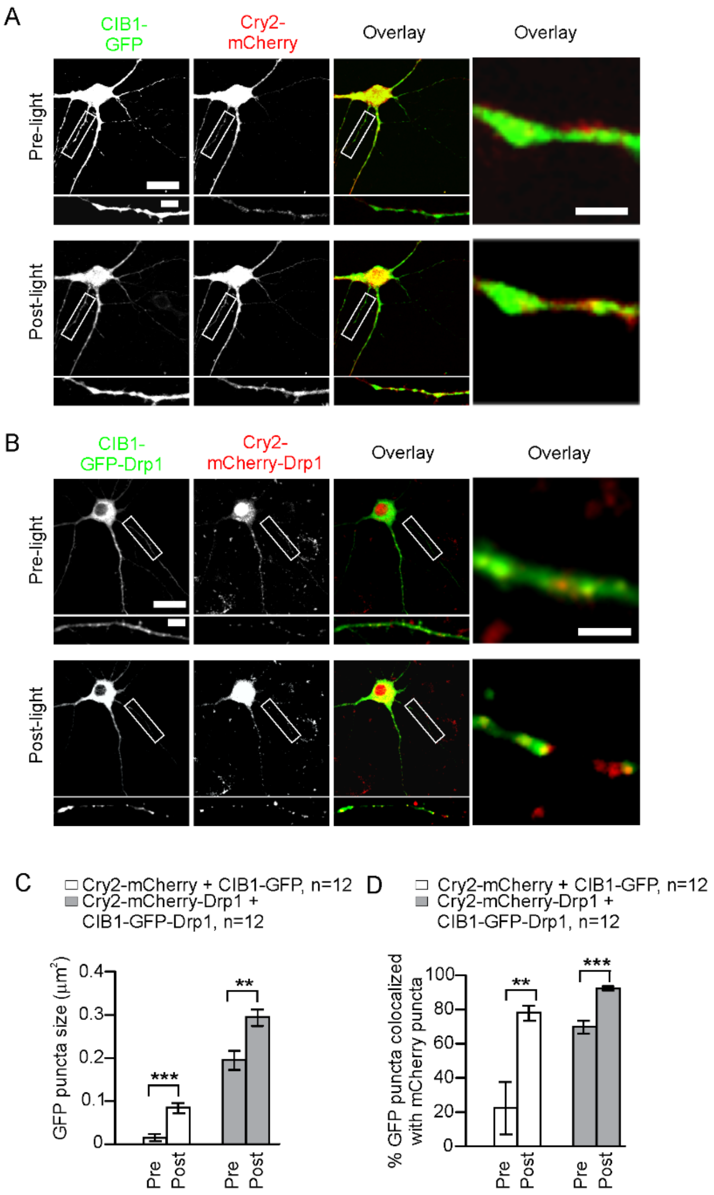

Supplemental Fig. 8. Light stimulation induces Drp1 oligomerization. Cultured hippocampal neurons were transfected with designated plasmids and imaged before and after stimulation with a 488-nm laser (1 mW/mm2, 10 min). (A, B) Representative images; scale bar, 20 μm for low-magnification images and 5 μm for high-magnification images. (C) The size of puncta positive for GFP; paired Student's t-test was used to compare same cells before and after light stimulation. (D) The proportion of GFP

positive puncta colocalized with mCherry; Wilcoxon signed rank test was used to compare same cells transfected with Cry2-mCherry and CIB1-GFP before and after light stimulation; paired Student's t-test was used to compare same cells transfected with Cry2-mCherry-Drp1 and CIB1-GFP-Drp1 before and after light stimulation. Data are presented as mean ± SEM; n indicates the number of neurons; ** $p < 0.01$, *** $p < 0.001$.

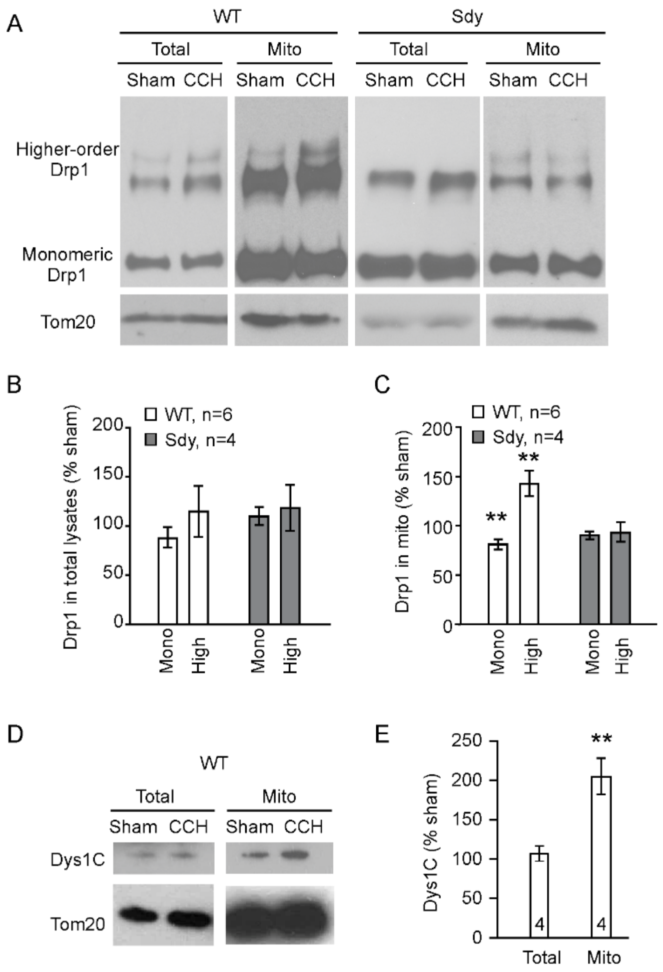

Supplemental Fig. 9. Carbachol induces dysbindin-1C translocation to mitochondria and increases higher-order Drp1 oligomers on mitochondria. Hippocampal slices from WT and sdy mice (7-8 weeks of age) were sham-treated or treated with carbachol (20 μM, 30 min), and then used for the preparation of total lysates and mitochondrial fraction. (A, D) Represent blots. (B, C) Quantification of monomeric Drp1 and higher-order Drp1 oligomers for A; One Way ANOVA on ranks was used to compare across groups for Drp1 in the total lysate ($H = 4.649$, $DF = 5$, $p = 0.460$) and mitochondrial fraction ($H = 20.884$, $DF = 5$, $p < 0.001$); Mann-Whitney U test was used to compare Drp1 monomer and higher-order structures between sham and oligomycin treated samples. (E) Quantification for D; Student's t-test was used to compare Dys1C

between sham and oligomycin treated samples. Data are presented as mean ± SEM; ** $p < 0.01$. n indicates the number of experiments.

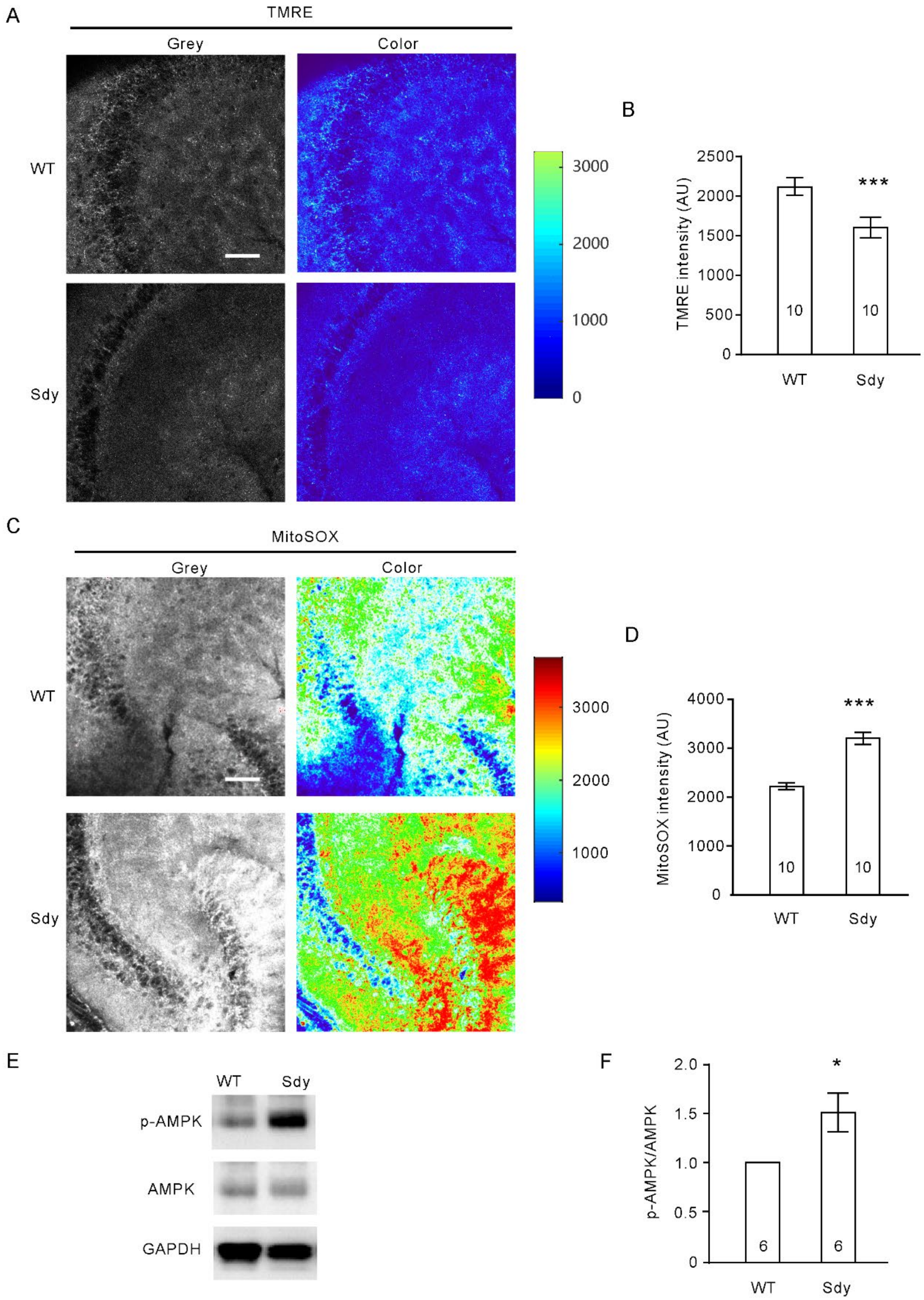

Supplemental Fig. 10. Mitochondrial membrane potential (MMP), reactive oxygen species (ROS) and cellular ATP are altered in sdy mice. (A–D) Hippocampal slices

were prepared from WT and sdy mice (7–8 weeks of age) and incubated with TMRE (100 nM, 20 min) or MitoSOX (5 μM, 10 min) followed by live-imaging. (A, C) Representative images in grayscale (left) and color scale (right) that indicate fluorescence intensity. (B) Quantification for A; Student's t-test was used for statistical analysis. (D) Quantification for C; Student's t-test was used for statistical analysis. (E, F) Total lysates were prepared from the hippocampus of WT and sdy mice (7–8 weeks of age) for immunoblotting. Mann-Whitney U test was used to compare the ratio of phosphorylated AMPK to total AMPK between WT and sdy samples. AU, arbitrary unit. Data are presented as mean ± SEM; n indicates the number of slices from 4 mice in B, D, and animal number in F; * $p < 0.05$, *** $p < 0.001$.

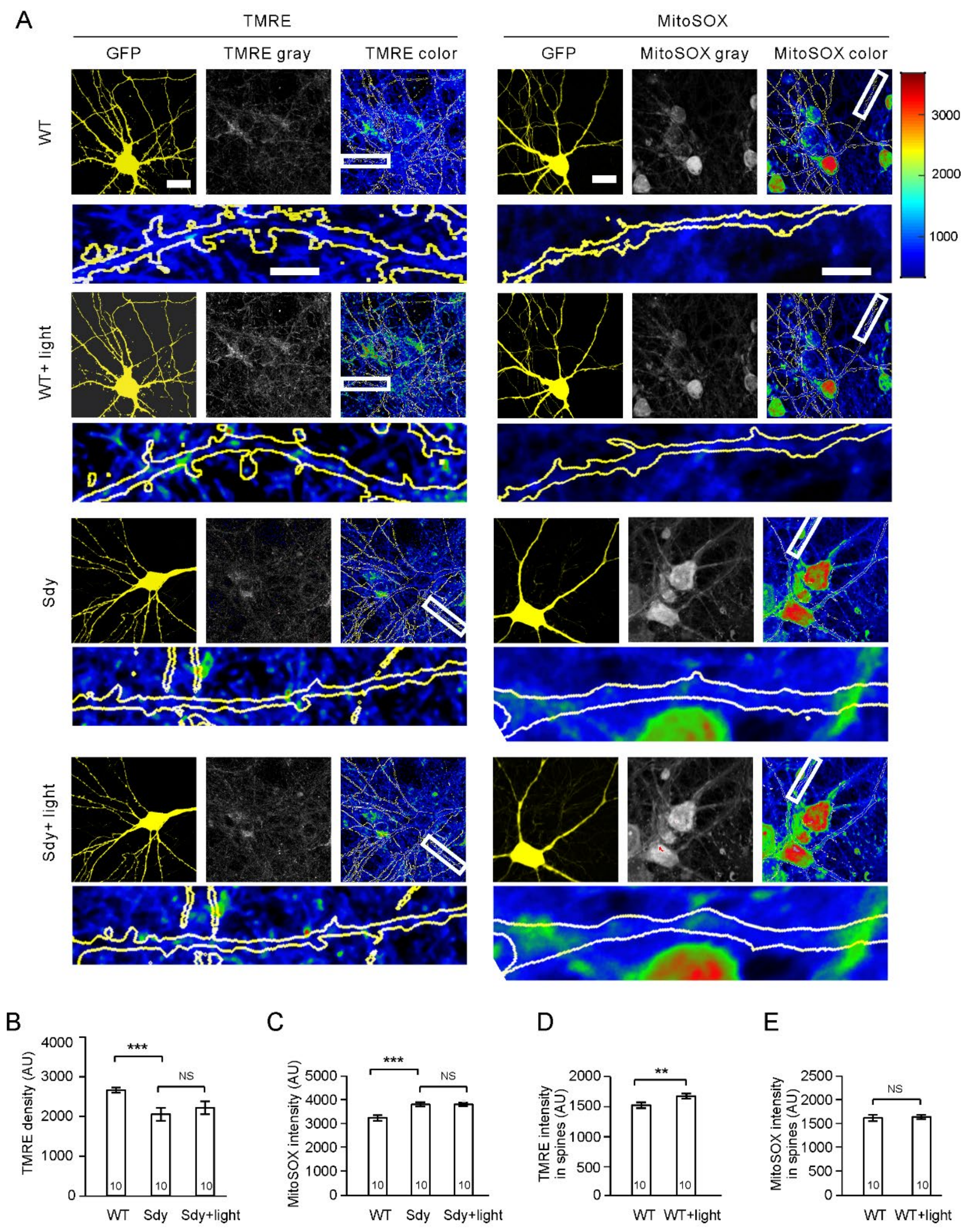

Supplemental Fig. 11. Light stimulation of Cry2-Drp1 and CIB1-Drp1 has no effect on MMP and ROS in sdy neurons. Primary hippocampal neurons from WT and sdy mice were transfected with Cry2-Drp1 and CIB1-Drp1, and stimulated with blue light (488 nm, 1 mW/mm^2) for 10 min. (A) Representative images in grayscale (middle) and color

scale (right) that indicate fluorescence intensity; scale bar, 20 μm for top and 5 μm for lower, higher magnification images. (B) Quantification for TMRE; one-way ANOVA was used to compare across groups, $F_{(2, 27)} = 7.589$, $p = 0.0024$. Student's t-test was used to compare WT and Sdy, paired Student's t-test was used to compare Sdy and Sdy + light. (C) Quantification for MitoSOX; one-way ANOVA was used to compare across groups, $F_{(2, 27)} = 9.855$, $p = 0.0006$. Student's t-test was used to compare WT and Sdy, paired Student's t-test was used to compare Sdy and Sdy + light. (D) Quantification for TMRE in spines; paired Student's t-test was used for statistical analysis. (E) Quantification for MitoSOX in spines; paired Student's t-test was used for statistical analysis. AU, arbitrary unit. Data are presented as mean ± SEM; n = 10 neurons for each group; ** $p < 0.01$, *** $p < 0.001$.
