# Supplementary figures and images for: Dysbindin-1 regulates mitochondrial fission and gamma oscillations
Source: Mol Psychiatry. 2021 Feb 15;26(9):4633–51. doi: 10.1038/s41380-021-01038-9 (PMC8364574; doi:10.1038/s41380-021-01038-9)

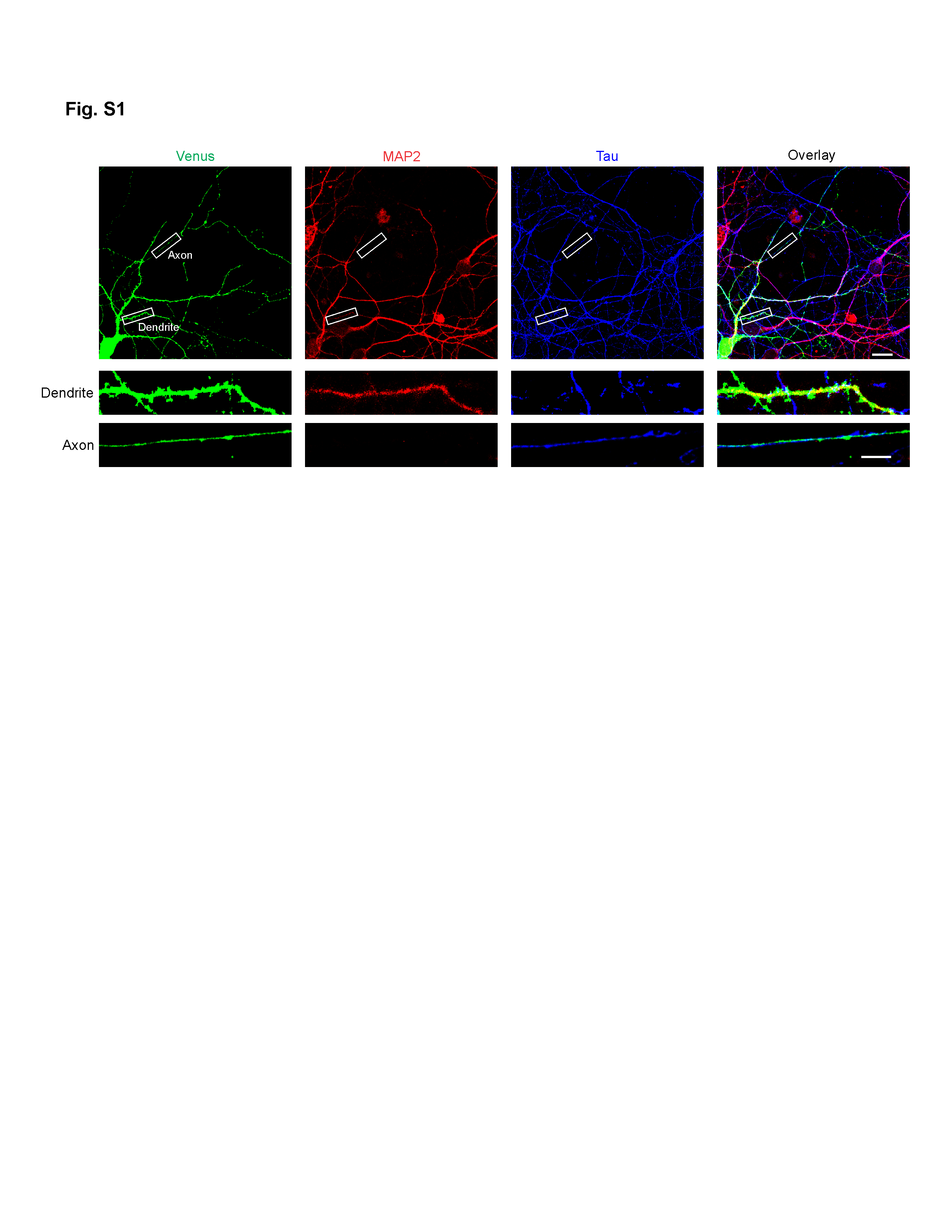

Supplement: Supplementary file 2 — Supplemental Figure 1 [file 41380_2021_1038_MOESM2_ESM.tif]

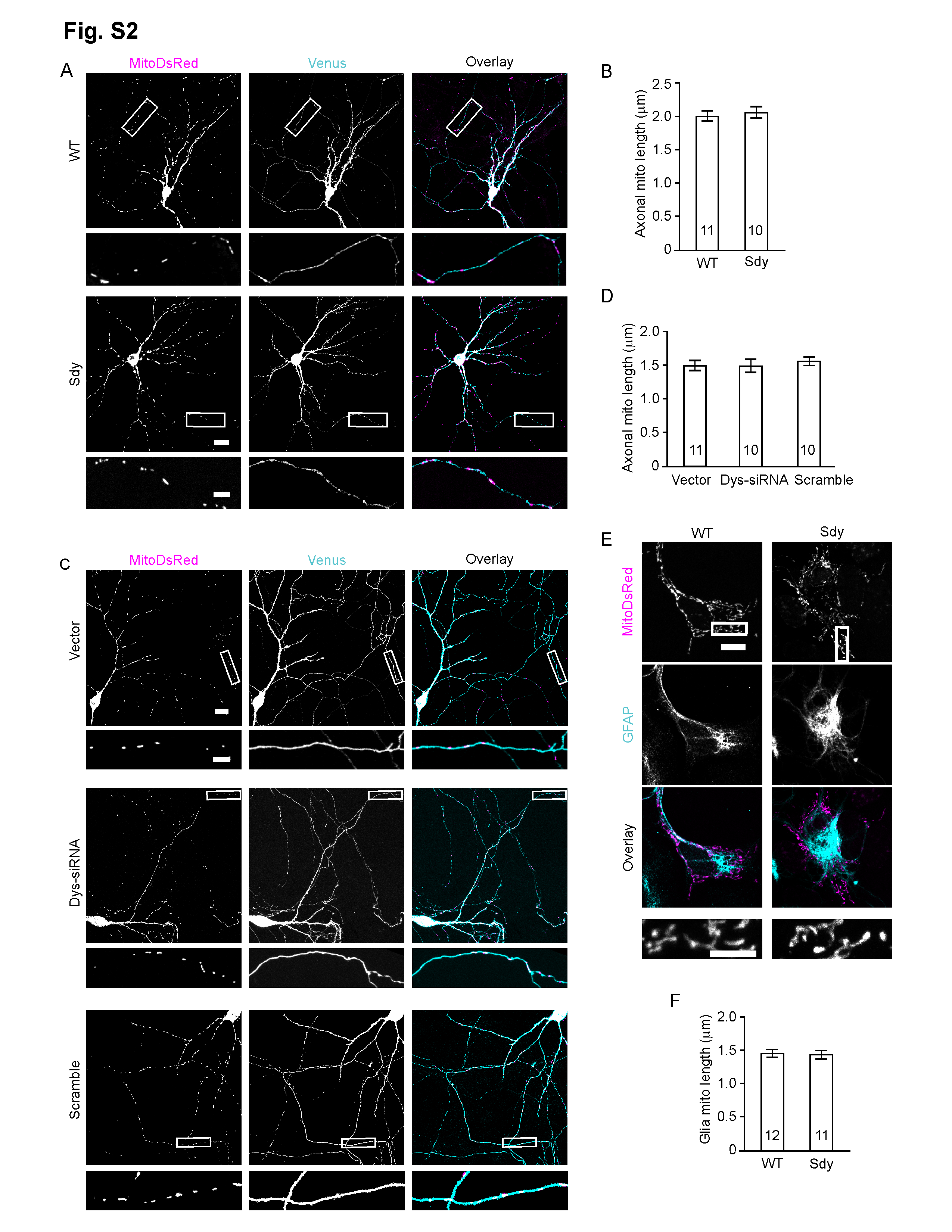

Supplement: Supplementary file 3 — Supplemental Figure 2 [file 41380_2021_1038_MOESM3_ESM.tif]

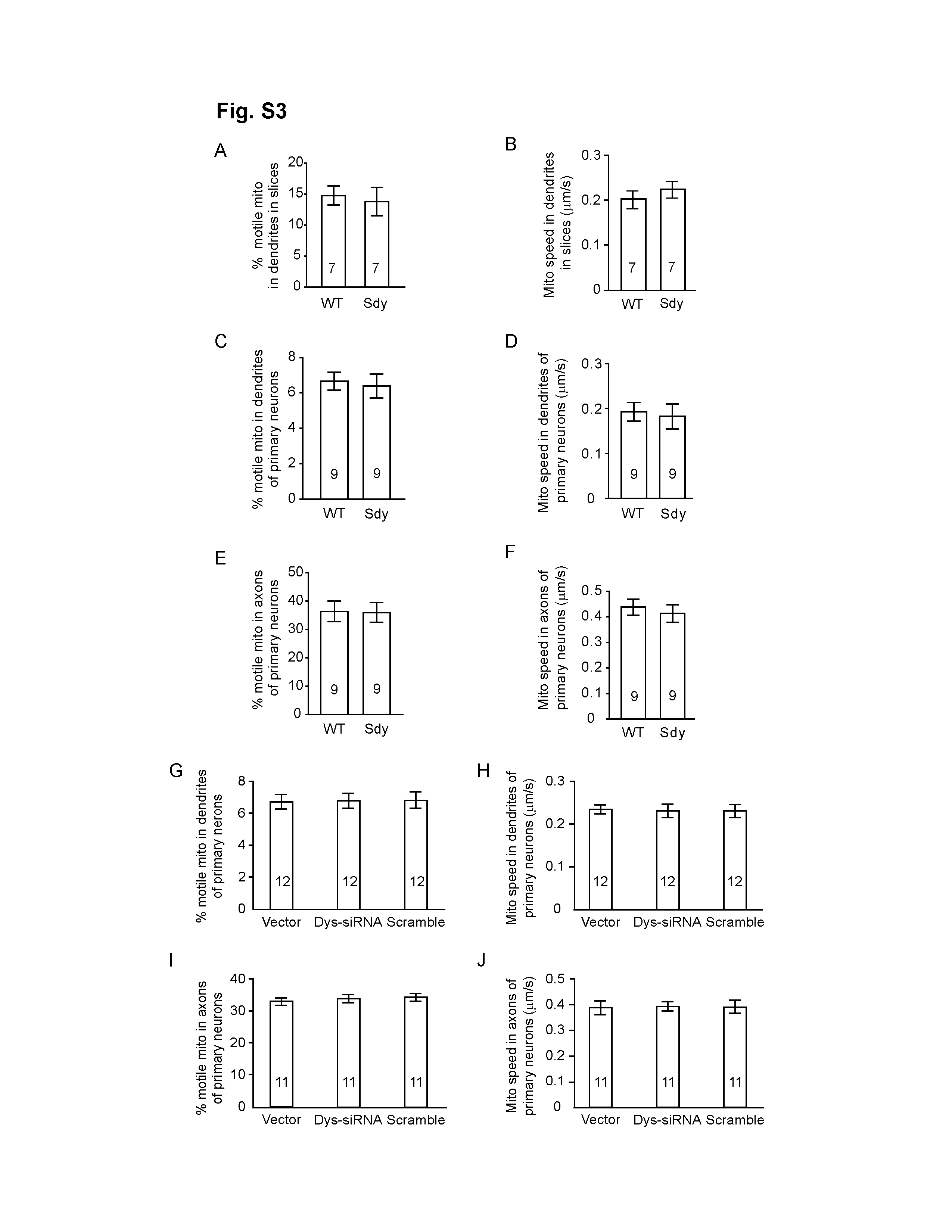

Supplement: Supplementary file 4 — Supplemental Figure 3 [file 41380_2021_1038_MOESM4_ESM.tif]

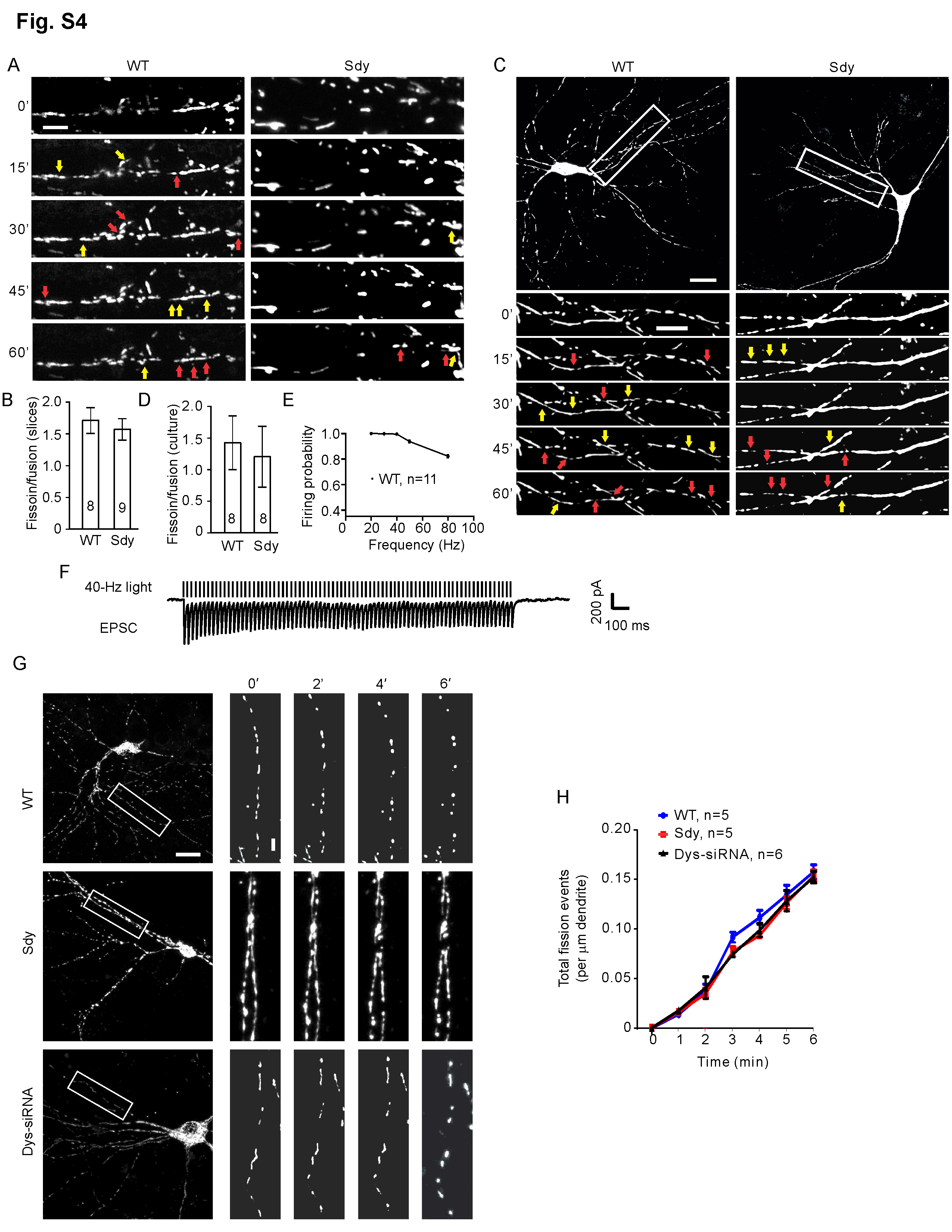

Supplement: Supplementary file 5 — Supplemental Figure 4 [file 41380_2021_1038_MOESM5_ESM.tif]

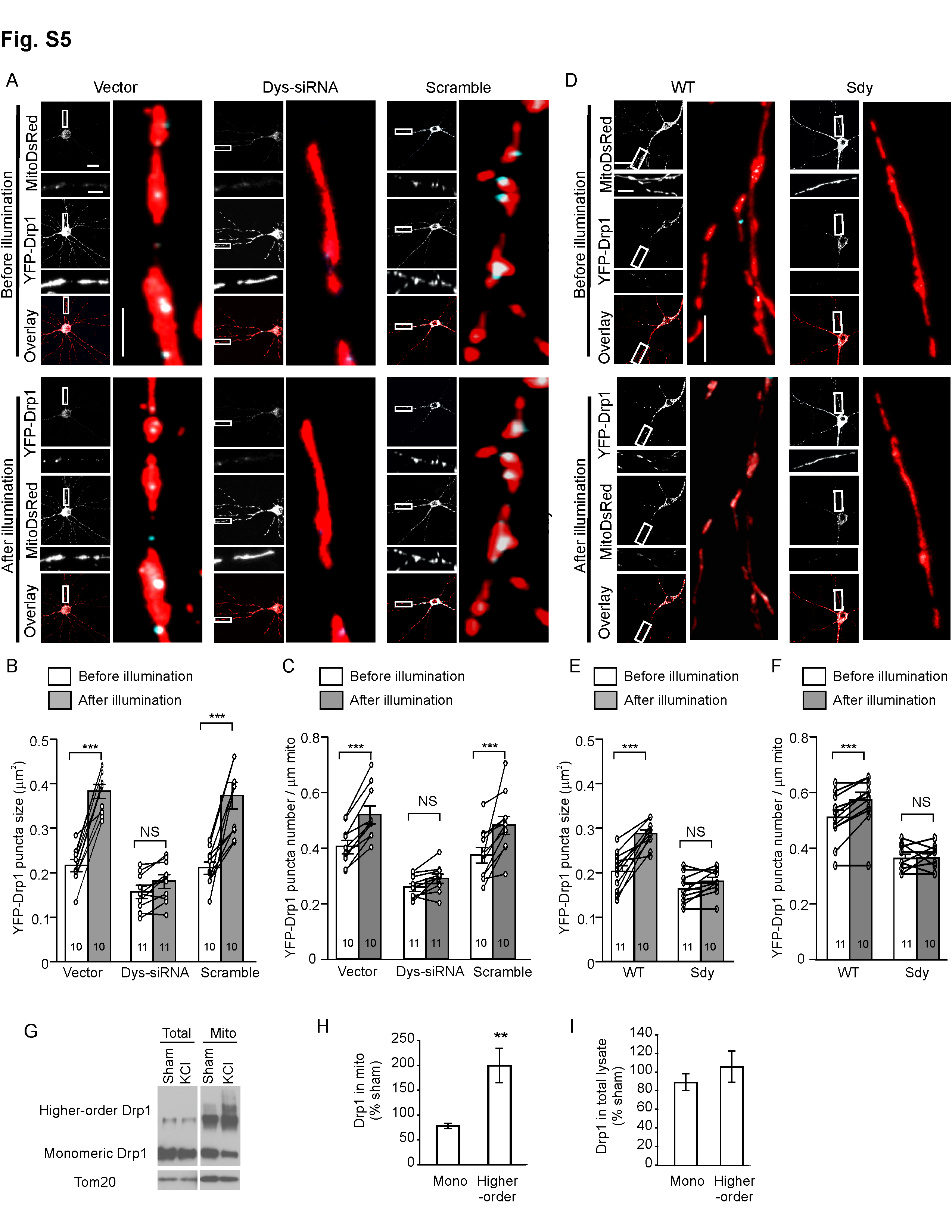

Supplement: Supplementary file 6 — Supplemental Figure 5 [file 41380_2021_1038_MOESM6_ESM.tif]

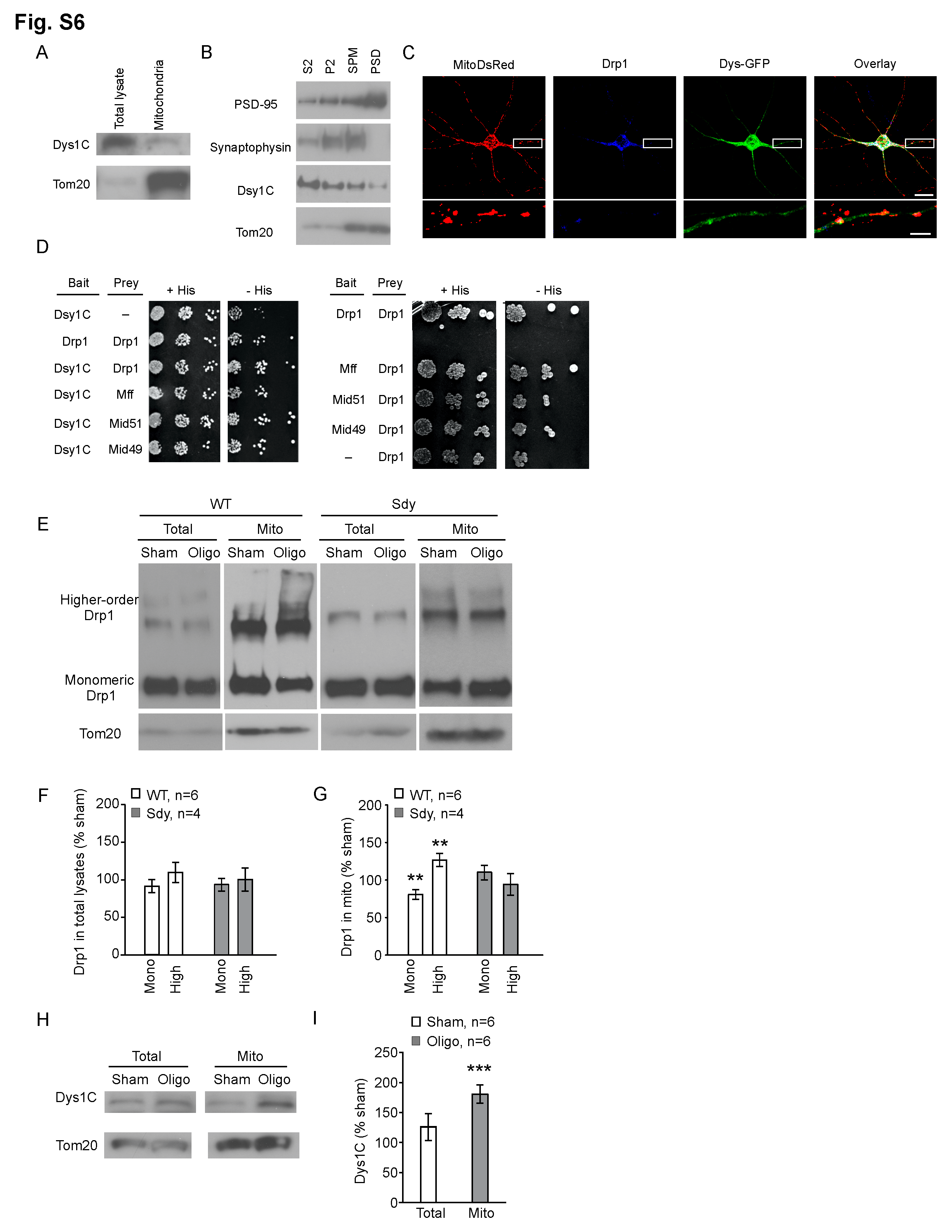

Supplement: Supplementary file 7 — Supplemental Figure 6 [file 41380_2021_1038_MOESM7_ESM.tif]

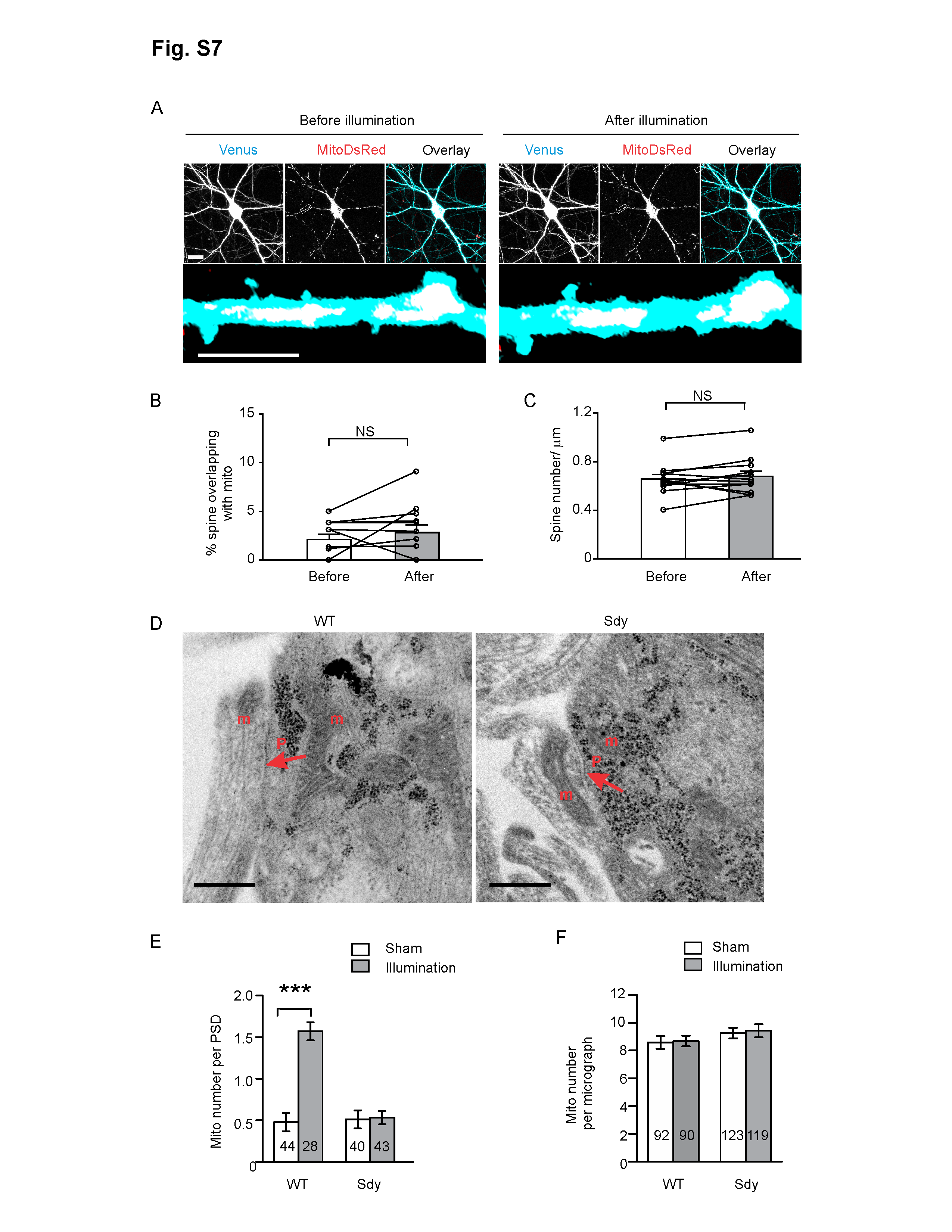

Supplement: Supplementary file 8 — Supplemental Figure 7 [file 41380_2021_1038_MOESM8_ESM.tif]

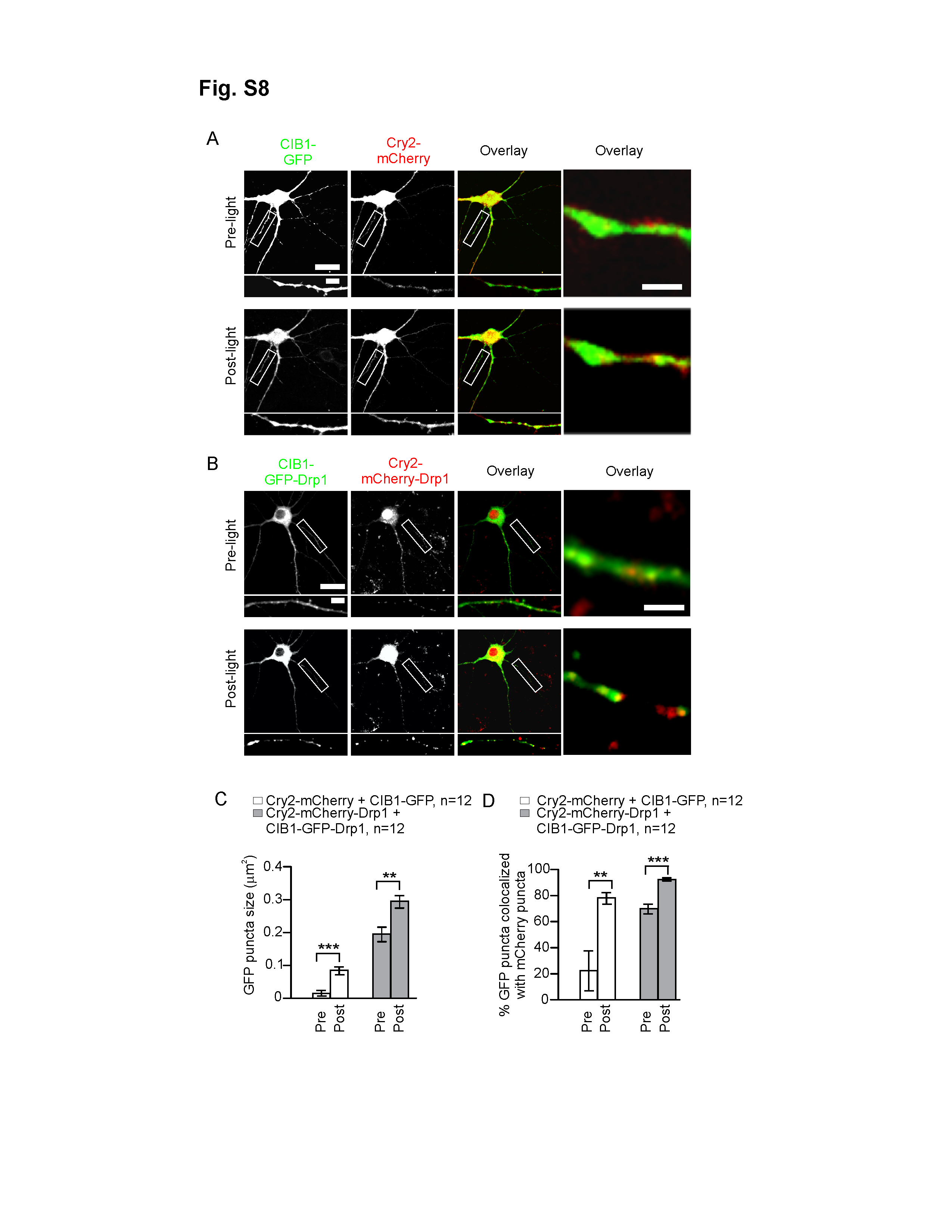

Supplement: Supplementary file 9 — Supplemental Figure 8 [file 41380_2021_1038_MOESM9_ESM.tif]

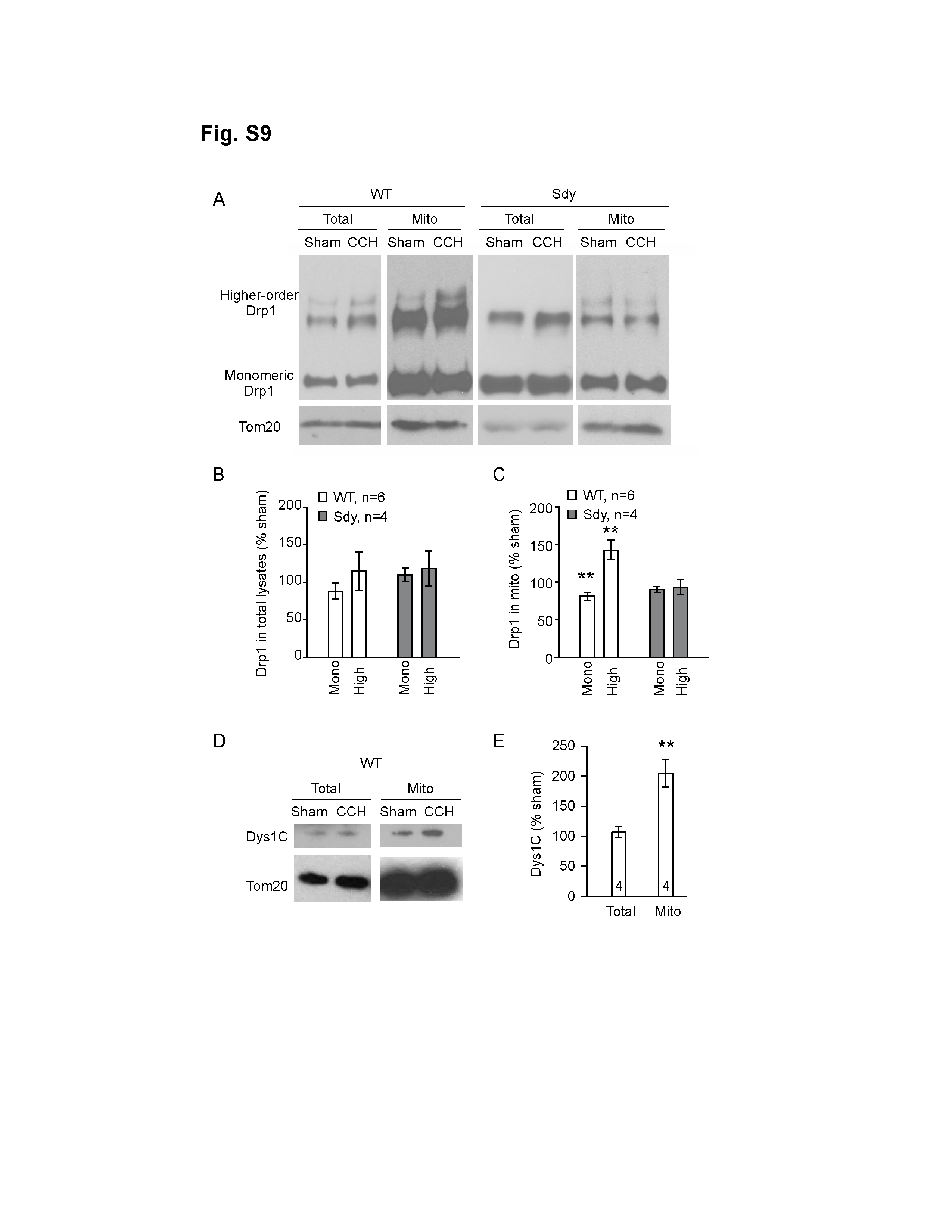

Supplement: Supplementary file 10 — Supplemental Figure 9 [file 41380_2021_1038_MOESM10_ESM.tif]

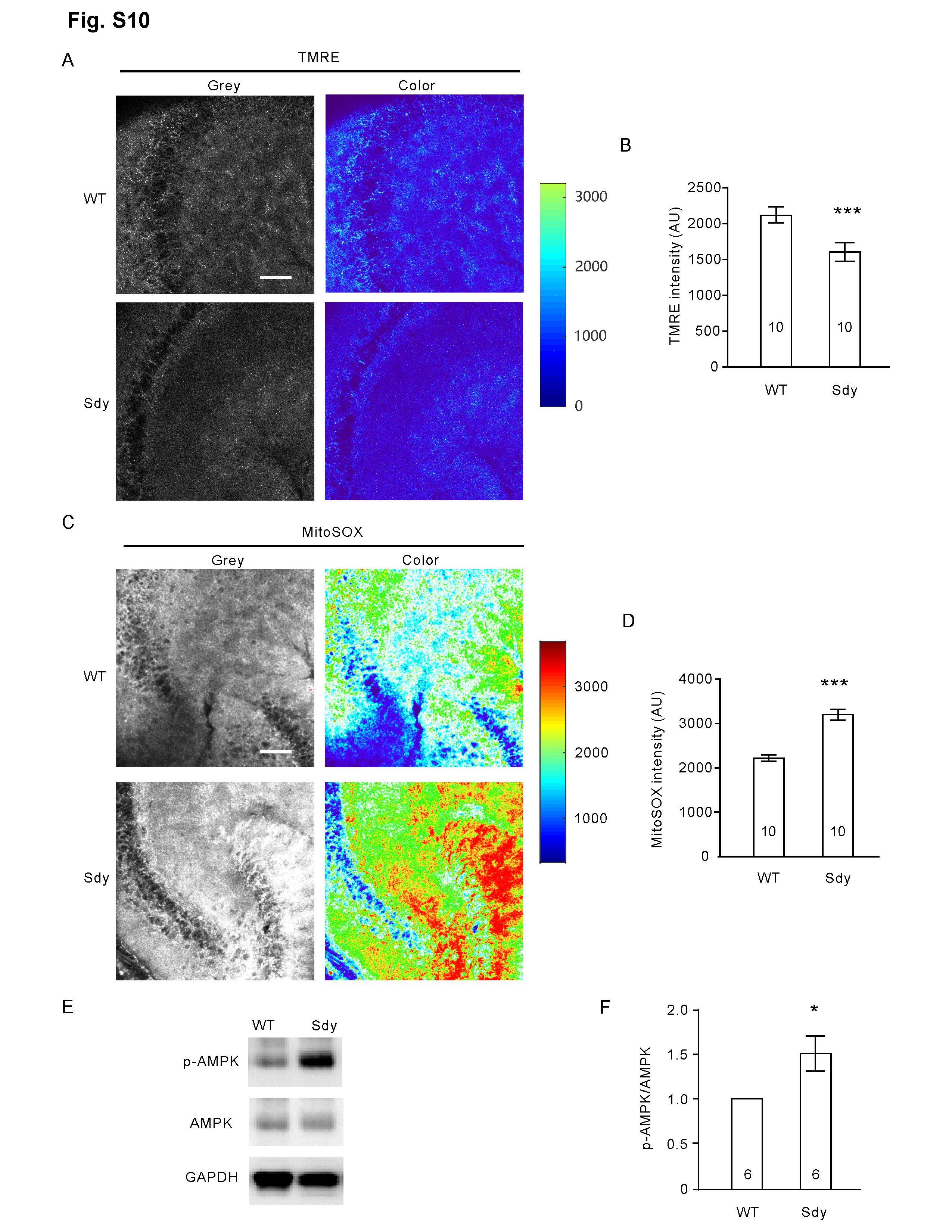

Supplement: Supplementary file 11 — Supplemental Figure 10 [file 41380_2021_1038_MOESM11_ESM.tif]

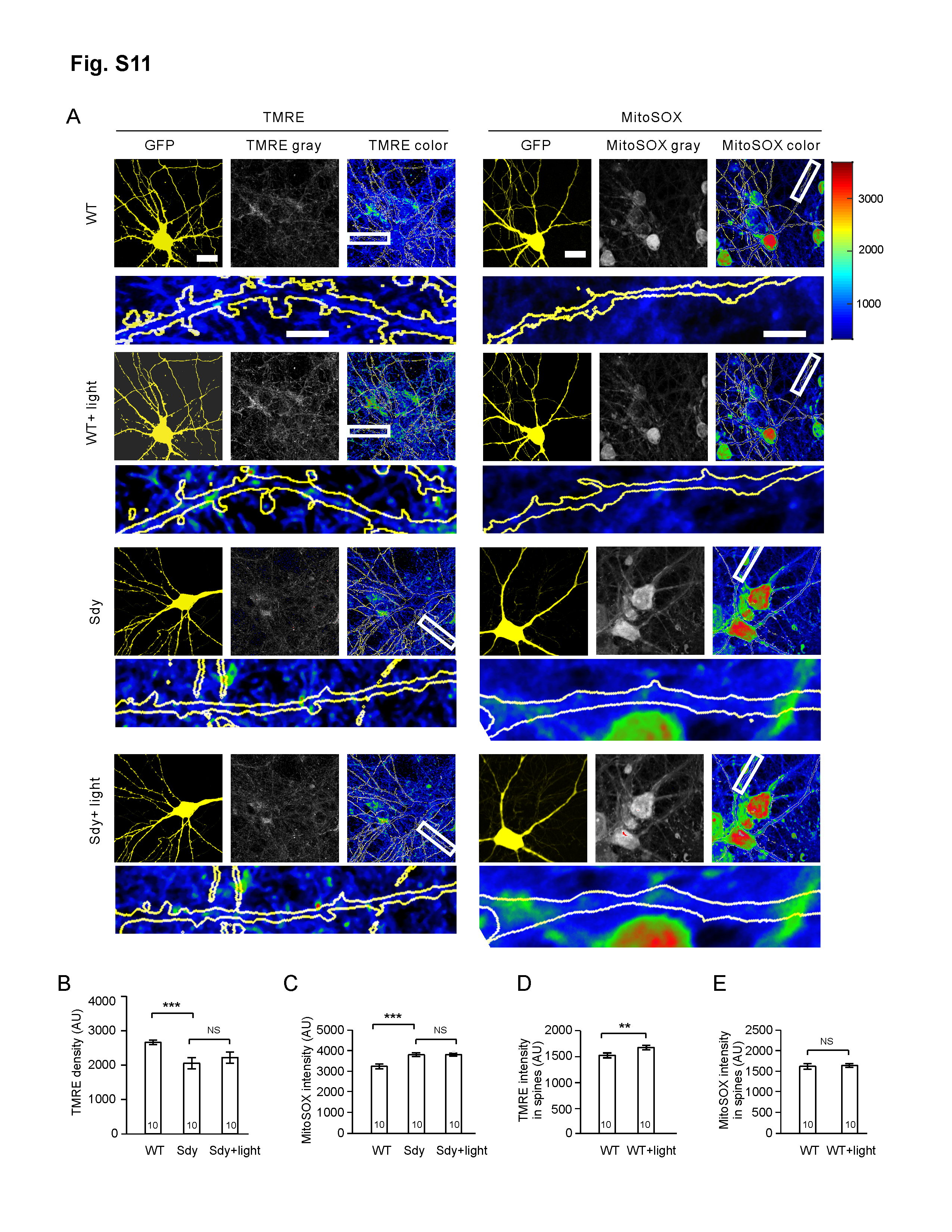

Supplement: Supplementary file 12 — Supplemental Figure 11 [file 41380_2021_1038_MOESM12_ESM.tif]
